# Association of RNAs with *Bacillus subtilis* Hfq

**DOI:** 10.1371/journal.pone.0055156

**Published:** 2013-02-15

**Authors:** Michael Dambach, Irnov Irnov, Wade C. Winkler

**Affiliations:** 1 Department of Biochemistry, The University of Texas Southwestern Medical Center, Dallas, Texas, United States of America; 2 Department of Cell Biology and Molecular Genetics, The University of Maryland, College Park, Maryland, United States of America; Loyola University Medical Center, United States of America

## Abstract

The prevalence and characteristics of small regulatory RNAs (sRNAs) have not been well characterized for *Bacillus subtilis*, an important model system for Gram-positive bacteria. However, *B. subtilis* was recently found to synthesize many candidate sRNAs during stationary phase. In the current study, we performed deep sequencing on Hfq-associated RNAs and found that a small subset of sRNAs associates with Hfq, an enigmatic RNA-binding protein that stabilizes sRNAs in Gram-negatives, but whose role is largely unknown in Gram-positive bacteria. We also found that Hfq associated with antisense RNAs, antitoxin transcripts, and many mRNA leaders. Several new candidate sRNAs and mRNA leader regions were also discovered by this analysis. Additionally, mRNA fragments overlapping with start or stop codons associated with Hfq, while, in contrast, relatively few full-length mRNAs were recovered. Deletion of *hfq* reduced the intracellular abundance of several representative sRNAs, suggesting that *B. subtilis* Hfq-sRNA interactions may be functionally significant in vivo. In general, we anticipate this catalog of Hfq-associated RNAs to serve as a resource in the functional characterization of Hfq in *B. subtilis*.

## Introduction

Regulation by small, trans-acting regulatory RNAs (sRNAs) is critical for bacterial gene regulation [1]. For example, there are approximately 100 sRNA regulators in *Escherichia coli* and *Salmonella* species [2], which contain a moderately similar number of transcription factors (∼200) affecting the efficiency of transcription initiation. Moreover, both classes of regulatory molecules (sRNAs and transcription factors) can influence the abundance of multiple target transcripts, indicating that genetic regulons can be controlled by both initiation and post-initiation regulatory strategies [3]–[4]. Therefore, it has become clear from these prior studies that sRNA-mediated regulation is an important second “layer” of genetic control.

In Gram-negative proteobacteria, sRNA regulators are expressed in response to certain stress or physiological conditions and are stabilized against degradation through association with the homohexameric RNA-binding protein Hfq [5]. Hfq is an Sm-like protein that is widespread in eubacteria [6]. For instance, most α-, β-, and γ-proteobacterial species encode at least one copy of *hfq*. Deletion or mutation of *hfq* results in decreased fitness and reduced virulence for many Gram-negative pathogens [7]. For example, deletion of *Salmonella typhimurium hfq* resulted in several phenotypic changes including but not limited to reduced replication in macrophages, incomplete secretion of virulence effectors, loss of motility, and attenuated virulence in mice [7]–[8]. It is generally presumed that Hfq’s importance stems almost entirely from its functions in sRNA-based regulation. In general, Hfq increases the intracellular half-life of sRNAs and facilitates base-pairing interactions between sRNAs and their mRNA targets. Hfq may assist sRNA-mRNA interactions by enhancing the rate of their annealing or by instigating RNA structural remodeling [9]–[12]. Consistent with this possibility, structural analyses of Hfq hexamers suggest that the ring-shaped complex exhibits at least two RNA-binding portions for these purposes [13].

Thus far, most sRNAs have been found to affect gene expression by binding to sites within 5' leader regions and altering translational efficiency of the downstream gene. However, some sRNAs associate within mRNA coding regions, while still other sRNAs control expression by altering mRNA stability [14]–[15]. Regardless, Hfq is required for virtually all sRNA-mediated gene regulation in *E. coli* and *Salmonella*. Correspondingly, multiple experimental methods have been employed to identify the full catalog of Hfq-associated sRNAs for several Gram-negative bacteria [16]–[17]. For example, one global study analyzed RNAs that co-immunoprecipitated with *E. coli* Hfq, using Hfq-specific antisera; the protein-associated RNAs were subsequently detected using high-density oligonucleotide microarrays [18]. Other studies have identified Hfq-associated RNAs through subcloning and sequencing of cDNAs [19]–[22]. More recently, the development of high-throughput sequencing methodologies has expanded the sensitivity and scale of these analyses [17]. To create a comprehensive catalog of sRNAs and mRNAs that co-immunoprecipitated with epitope-tagged Hfq in *Salmonella*, high-throughput pyrosequencing was used to identify the protein-associated RNAs [23]. This method successfully recovered known *Salmonella* sRNA genes and led to the discovery of new sRNAs, raising the total number of sRNAs in this organism to greater than 100. When Hfq-associated mRNAs were examined against the transcriptomic profile of an *hfq* mutant strain, these data also allowed for the preliminary prediction of global mRNA targets. In general, the high-throughput sequencing-based approach is rapid, global, and exhibits a wide dynamic range for quantification of protein-associated RNA species.

In contrast, less is known regarding the relative importance of sRNAs in Gram-positive bacteria. Similarly, the role(s) of Hfq in Gram-positive bacteria have also been incompletely characterized [24]. In *Staphylococcus aureus*, at least a dozen sRNAs have been identified, whose intracellular abundance is independent of Hfq, in contrast to the general requirement for Hfq by proteobacteria [25]–[26]. Most of the *S. aureus* sRNAs included a C-rich motif that is likely to be important for association with their target mRNAs. Interestingly, one of these sRNAs, coined RsaE, was found to be conserved in other Bacillaceae, including *B. subtilis*, and is predicted to regulate expression of metabolic genes through the C-rich motif [25], [27]. The most thoroughly studied *S. aureus* sRNA, RNAIII, utilizes an Hfq-independent mechanism to regulate expression of multiple mRNAs involved in virulence. Therefore, it is still unclear what role(s) Hfq might play in this organism. *Streptococcal* species, which do not encode a known Hfq homologue, have also been found to produce multiple putative sRNA regulators [24]. Similarly, over 50 putative sRNAs have been identified in *Listeria monocytogenes* [19], [28]; of these, a single sRNA, LhrA, associates with its target mRNA in an Hfq-dependent manner [22]. Therefore, it is still unclear what, if any, role Hfq plays with sRNA-mediated regulation in these bacteria. The fact that Hfq does not appear to be required or associate with sRNAs from these bacteria has suggested that it may not play a general role in sRNA regulation. Perhaps, the sRNA-mRNA interactions within these bacteria are thermodynamically sufficient without Hfq; however, this hypothesis remains to be fully explored. Alternatively, other RNA chaperones may be redundant with Hfq in these organisms, effectively masking a functional requirement for protein-mediated assistance of sRNA-mRNA interactions. Indeed, some evidence supports the latter hypothesis. Specifically, several small proteins have been shown to assist posttranscriptional regulation by the *B. subtilis* sRNA, FsrA [29]–[30]. Despite the lack of functional evidence for a role in RNA metabolism, Hfq variants from multiple Gram-positive bacteria have been analyzed by X-ray crystallography. These data together have shown Hfq to associate with RNAs in vitro [31]–[32]. To further explore the potential functional roles for Hfq in Gram-positive bacteria, we investigated in this study whether RNA molecules associated with Hfq for the model microorganism, *B subtilis*.

Many (>150) putative sRNAs have been identified in *B. subtilis* [27], [33]–[35]. However, very few of these putative regulatory RNAs have been characterized, either in regards to their target mRNA(s) or mechanism of action. One such sRNA, called RatA, is an antisense sRNA that associates with a toxin-encoding mRNA, *txpA*, and is believed to decrease expression of the toxic peptide [36]. At least three other so-called type I toxin-antitoxin systems have been identified in this bacterium [33], [37]. Another cis-encoded antisense RNA has been found to base pair with an uncharacterized gene, *yabE* [38]. Three sRNAs have been characterized to varying degrees with respect to their regulatory functions: SR1, FsrA, and RsaE. SR1 has been demonstrated to control expression of *ahrC*, which encodes a transcriptional activator of arginine catabolism genes [39]. FsrA is expressed upon derepression of the iron-regulatory protein, Fur, and affects production of iron-containing proteins [29]. Finally, RsaE, an unusually widespread sRNA in diverse Gram-positive species, has been predicted to target central metabolism genes and a ‘carbon starvation’ gene, *cstA* [25], [27]. Notably, the potential role for RNA chaperones, such as Hfq, has not been fully explored for these sRNAs. Indeed, it was not known whether Hfq (or other RNA-binding proteins) affect the abundance of any *B. subtilis* sRNAs, or even if it was expressed under standard bacterial growth conditions. In this study, we find that Hfq is expressed in minimal and rich media, but that expression is increased during stationary phase conditions. To identify potential RNA ligands for association with Hfq we incorporated an epitope-tagged copy of *hfq* into the genome and co-immunoprecipitated Hfq from stationary phase cells. Protein-associated RNAs were converted to cDNA and then identified using high-throughput sequencing. We find that *B. subtilis* Hfq broadly associates with different classes of RNA molecules in vivo, including portions of mRNAs, numerous 5' leader regions, and approximately 25% of the putative sRNAs that were identified previously. Mutational disruption of *hfq* reduced the steady state abundance for a subset of the sRNAs, suggesting that the Hfq-sRNA interactions predicted by co-immunoprecipitation may be important in vivo. These data demonstrate that *B. subtilis* Hfq broadly associates with RNA molecules and correspondingly may influence regulatory RNA networks.

## Results and Discussion

### Expression of *hfq* in *Bacillus subtilis*

Hfq has yet to be examined for *Bacillus subtilis*, a model microorganism for the low-GC Gram-positive bacteria. In *Staphylococcus aureus*, the putative *hfq* gene is only weakly expressed [40] and is not required for stabilization or regulation of any of the identified *S. aureus* sRNAs [24]. Interestingly, the Hfq sequences for many Gram-positive bacteria appear to lack a short, positively-charged stretch of amino acids at the C-terminus. For example, the *B. subtilis hfq* gene (originally annotated as *ymaH*) encodes a 73 amino acid Hfq homolog while, in contrast, *E. coli* Hfq contains an additional 29 amino acids at its C-terminus (Fig. S1). Indeed, the absence of this region has been proposed to be a key functional limitation for Hfq in many Gram-positive species [41]. However, recent data demonstrated that an *E. coli* Hfq containing a truncation of this C-terminal extension was still proficient in sRNA-mediated regulation [42]. Some bacteria (*e.g., Moraxella catarrhalis* and *Acinetobacter baylyi*) contain even larger C-terminal regions [43]–[45]. Therefore, the general importance and functional roles of the C-terminal portion of Hfq are currently unclear. It is therefore possible that *B. subtilis* Hfq may share functional similarities with its Gram-negative Hfq counterparts, despite the lack of the C-terminal extension.

**Figure 1:**
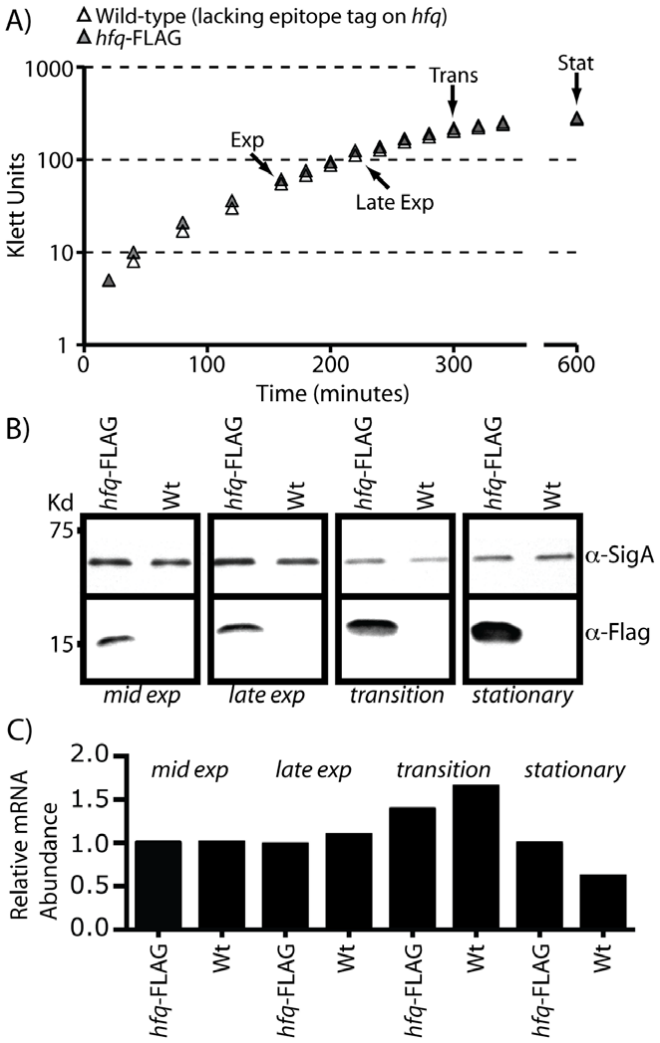
Expression of *hfq* in *B. subtilis*. Growth of wild-type *B. subtilis* 168 and *hfq* ^FLAG^ in minimal medium. Cell density was measured at 20 minute intervals using a Klett meter. Arrows denote the time points where samples were collected for analysis by Western blotting and quantitative real-time RT-PCR (qPCR). (B) Whole cell lysates were resolved on a 4–20% SDS-PAGE gel and transferred to a nitrocellulose membrane. Hfq^FLAG^ was detected using α-FLAG monoclonal antibody and visualized by chemiluminescence. The blots were stripped and re-probed with α-Sigma A rabbit serum as a loading control. (C) Measurement of *hfq* transcript abundance by qPCR. The experimentally determined *C*t values were normalized both to the corresponding value obtained at mid exponential phase and to that of *sigA*. Values are reported as the fold change in expression, relative to mid-exponential growth phase.

In many bacteria, *hfq* is co-transcribed with an adjacent tRNA modification enzyme, *miaA*. Indeed, *miaA* is located immediately upstream of *hfq* in the *B. subtilis* genome, suggesting a similar arrangement in this bacterium (Fig. S1). However, we previously used a high-throughput sequencing method to identify transcription start sites (TSS) for *B. subtilis* during stationary phase growth [33] and investigation of the *hfq* locus reveals a single start site located upstream of *hfq* and downstream of *miaA* (Fig. S1); therefore, *B. subtilis hfq* is likely to be expressed as a monocistronic transcript. Next we cultured cells in rich and minimal media and extracted total RNA at varying points during growth (Fig. 1). The relative abundance of the *hfq* transcript was determined by quantitative real-time RT-PCR (qPCR) (Fig. 1). Very little (<2-fold) change in mRNA abundance was observed as bacteria transitioned from exponential to stationary phase growth, suggesting that *hfq* is not likely to be transcriptionally regulated. To investigate whether protein levels were altered under these conditions, we replaced the endogenous *hfq* with an epitope (FLAG)-tagged copy and monitored protein abundance by Western blot analysis (Fig. 1). Interestingly, Hfq production was significantly increased upon transition to stationary phase growth. Therefore, we postulate that the *hfq* gene is likely to be subjected to either translational or post-translational regulation, although the exact mechanism remains to be identified. Overall, these data demonstrated that Hfq is produced in *B. subtilis*, and that its function is most likely relevant during stationary phase conditions; therefore, these conditions were selectively chosen for subsequent analyses.

### General Approach for Identifying Hfq-associated RNAs

In *Listeria monocytogenes*, Hfq has been reported to play a role in stress tolerance and pathogenesis in mice and has been shown to facilitate association of an antisense sRNA to its corresponding mRNA target [22], [46]. However, a broad role for Hfq has still yet to be determined in other Gram-positive bacteria, even though it is present in approximately 50% of sequenced genomes and contains conserved residues that are important for RNA binding activity. In an effort to better understand the function of Hfq in *Bacillus subtilis*, we co-immunoprecipitated FLAG-tagged Hfq and employed massively parallel sequencing for identification of protein-associated RNA molecules (Fig. S2). A wild-type strain lacking the epitope tag was used as a negative control. The cells were cultured to stationary phase in defined medium prior to preparation of cellular extracts; these conditions were chosen based upon the expression pattern of Hfq.

**Figure 2:**
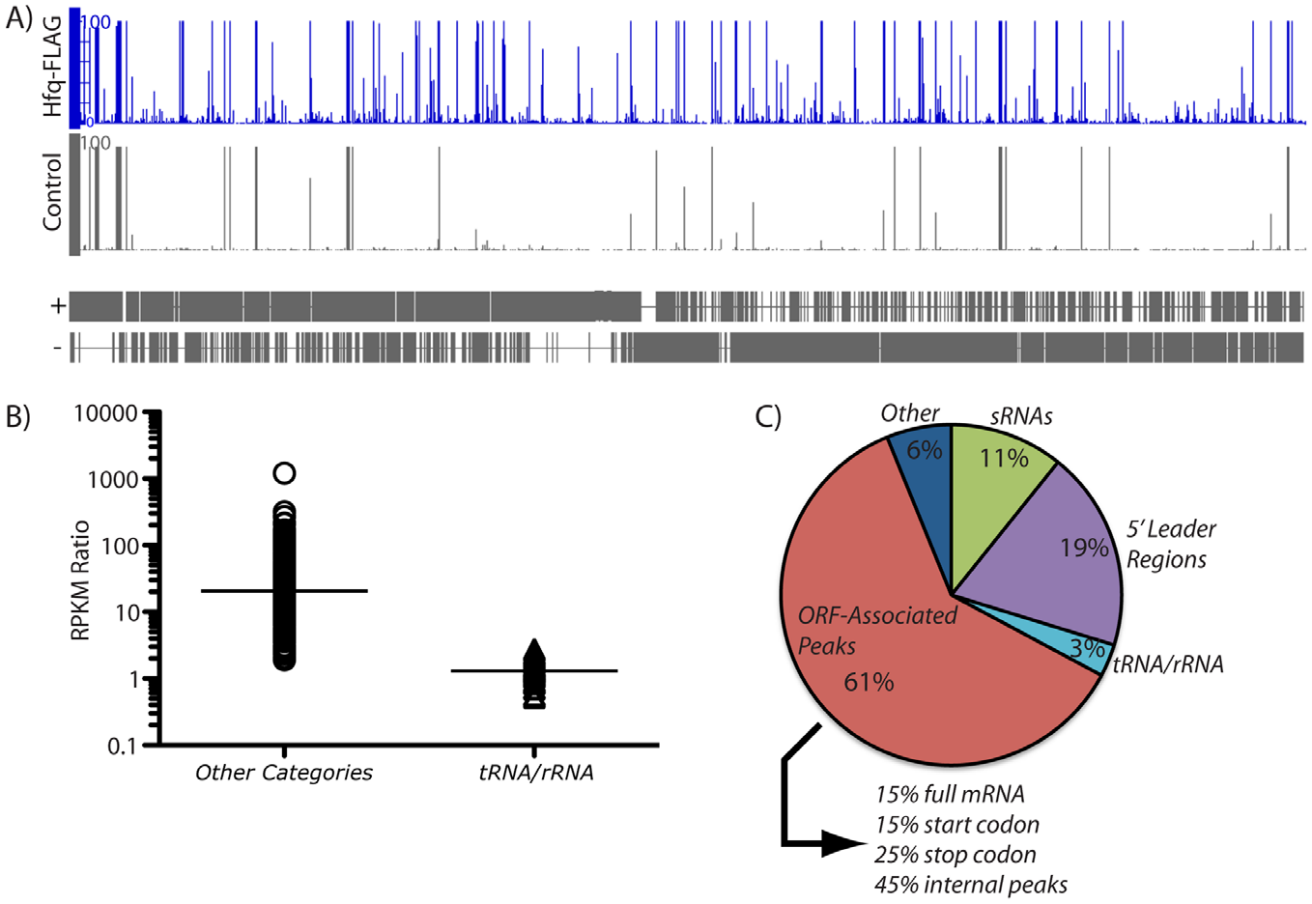
Co-immunoprecipitation of RNAs that associate with *Bacillus subtilis* Hfq. (A) Hfq^FLAG^ was co-immunoprecipitated from cellular extracts of stationary phase cells using an α-FLAG monoclonal antibody. A cDNA library was then created from the Hfq-associated RNA molecules, which was subjected to high-throughput sequencing using an Illumina Genome Analyzer. The cDNA peaks that resulted from this analysis were evenly distributed across the genome for both the Hfq^FLAG^ and mock control samples. Many more peaks were obtained from the Hfq^FLAG^-associated material. (B) The expression of each peak was quantified in reads per kilobase per million mapped reads, or ‘RPKM’ [47]. The ratio of these values for the Hfq^FLAG^ and mock control samples was calculated in order to identify the peak subset that was enriched by association with Hfq. Almost all peaks that exhibited an RPKM ratio of <2 (median  = 1.2) corresponded to “housekeeping” RNAs, such as tRNAs, rRNAs, and the RNA subunit of RNase P (see Table S1 for details). The remaining peaks exhibited a range of RPKM ratios (median  = 20). (C) The majority of the Hfq-enriched peaks (exhibiting an RPKM >2) corresponded to portions of open reading frames (ORFs). Smaller subsets of peaks corresponded to 5' leader regions and putative small RNAs (sRNAs).

Illumina-based sequencing of cellular RNAs that were recovered from coIP of Hfq resulted in approximately 25 million and 10 million total cDNA reads for the Hfq-FLAG and control samples, respectively. The average length of the cDNA sequences in this experiment was approximately 35 nucleotides. Approximately 10 million and 1.5 million cDNA reads were unambiguously matched to the *B. subtilis* reference genome for the Hfq-FLAG and control samples, respectively. Approximately 98% of the unmapped cDNA reads corresponded to the adapter oligonucleotides that are employed for Illumina sequencing. To identify clusters of cDNA reads corresponding to cellular RNAs we searched for peaks that exhibited at least five cDNA reads for a minimum of 50 consecutive genomic positions. RPKM values (cDNA reads per kilobase of genomic sequence per total mapped reads; [47]) were determined for all peaks identified from the Hfq-FLAG and control samples, and used to estimate the degree of enrichment by Hfq, which ranged from <1 to >1,000 (Fig. 2B; Table 1–2; Table S1, S2, S3). Almost all of the peaks exhibiting an RPKM ratio of less than two corresponded to tRNAs or rRNAs (Fig. 2B), suggesting that those RNA molecules were not likely to be enriched by co-immunoprecipitation with Hfq under our experimental conditions. Based in part on this observation, we applied an RPKM ratio of two as an arbitrary cut-off for more detailed analysis of the remaining coIP peaks.

**Table 1:** Putative sRNAs that coIP with Hfq.

Annotation	Peak Length	Peak Coord	Left-Right Genes	RPKM Ratio^a^	Ref
***bsrC***	146	474265–474410	*ydaG-ydaH*	53.6	[73]
***bsrE/ncr1857*** d	275	2069840–2070114	*yoyA-yobJ*	50.9	[33]
***shd60*** b	87	2190671–2190757	*Anti-yoqZ*	48.2	[27]
***ncr982***	81	1917500–1917580	*fosB-lexA*	47.7	[33]
***ncr952***	106	1780422–1780527	*mutL-ymzD*	45.0	[33]
***ncr1430***	50	4035605–4035654	*bglP-yxxE*	36.6	[33]
***ncr3000*** b,d	103	2219742–2219844	*Anti-yonT*	35.7	New
***RatA*** d	199	2678350–2678548	*Anti-txpA*	33.5	[33]
***ncr3001***	125	972865–972989	*yhbF-prkA*	24.4	New
***csfG***	136	1569212–1569347	*ylbG-ylbH*	22.9	[62]
***ncr2184/ncr60***	234	2779115–2779348	*yrzI-yrhG*	22.2	[27], 33
***ncr2166/as-bsrH*** d	301	2678750–2679050	*yqdB-yqbM*	20.7	[33]
***ncr1932/as-bsrG*** d	311	2273552–2273862	*yolA-yokL*	16.4	[33]
***ncr560/ncr18***	217	1056393–1056609	*yhaZ-yhaX*	13.9	[27], [33]
***FsrA***	73	1483560–1483632	*ykuI-ykuJ*	11.1	[29]
***ncr3002*** c	80	1447035–1447114	*ykzR-ykvR*	11.1	New
***SR2/BsrF***	93	2079102–2079194	*yobO-csaA*	7.75	[33], [59]
***RsaE***	83	1233446–1233528	*yizD-yjbH*	3.97	[25], [27], [33]
***ncr471***	151	820667–820817	*yfmI-yfmG*	3.56	[33]
***ncr3003***	101	1609113–1609213	*ylmC-ylmD*	3.09	New
***ncr1670***	256	1077037–1077292	*hinT-ecsA*	2.77	[33]
***ncr1015***	75	2054012–2054086	*pps-xynA*	2.35	[33]

a The expression of each peak (described in the main text) was quantified in reads per kilobase per million mapped reads, or ‘RPKM’ [47]. The ratio of these values for the Hfq^FLAG^ and mock control samples was taken as an indicator of Hfq-mediated enrichment.

b These sRNAs are arranged such that they appear to consist of putative antisense transcripts.

c This peak is adjacent but not overlapping with the previously identified sRNA candidate, ncr721/ncr34 [27], [33].

d These peaks correspond to previously identified type I toxin:antitoxin systems [33], [37].

**Table 2:** 

Annotation	Peak Length	Peak Coord	Genes	RPKM Ratio^a^
New sRNA or *yrhE* leader region^b^	139	2781029–2781167	*yrhF-yrhE*	170
New sRNA or between *xre-xkdA* d	87	1321203–1321289	*xkdA-xre*	150
*srfAA* leader region^f^	150	376704–376853	*hxlR-srfAA*	134
*sdhC* leader region^g^	87	2908722–2908808	*sdhC-yslB*	106
New sRNA or *yybS* leader region^b,c^	92	4166607–4166698	*yybS-yyzH*	86.1
Uncharacterized *ypzK* leader region	54	2427780–2427833	*ypzK-ribH*	75.3
Uncharacterized *guaA* leader region^b,c^	125	692572–692696	*yebA-guaA*	73.2
Uncharacterized *yqhQ* leader region	171	2540947–2541117	*yqhR-yqhQ*	23.2
Unknown; Possibly a 3′ UTR for *folD*	69	2528316–2528384	*yqiB-folD*	22.1
Conserved RNA in the *dagK* 3′ UTR^e^	112	737436–737547	*dagK-yefA*	18.1
Unknown or *speD* leader region^b^	79	2966841–2966919	*speD-gapB*	14.1
Uncharacterized *yxjB* leader region^b,c^	174	4005159–4005332	*yxjB-yxjA*	7.05

a The expression of each peak (described in the main text) was quantified in reads per kilobase per million mapped reads, or ‘RPKM’ [47]. The ratio of these values for the Hfq^FLAG^ and mock control samples was taken as an indicator of Hfq-mediated enrichment.

b, c A previous report suggested that these genes have a leader region which encompasses the Hfq CoIP peaks reported herein [27], [33].

d This peak appears to correspond to either a previously unidentified sRNA candidate, or a portion of the intercistronic region between *xre* and *xkdA*.

e A conserved RNA element was discovered within the 3′ UTR of several transcripts in *B. subtilis*, including *dagK*. The function of this RNA structural element remains unknown [27].

f The transcription start site of *srfAA* was determined previously [74].

g The transcription start site of *sdhC* was determined previously [75].

One potential disadvantage of the method we used for Illumina sample preparation was that information regarding the genomic strand from which an RNA species originated was lost upon conversion to cDNA. However, our lab previously used a different high-throughput sequencing method for identification of approximately 600 *B. subtilis* transcription start sites (TSS) [33]. Importantly, the culture conditions for the prior TSS analysis were identical to the culture condition that was used for the Hfq coIP experiment described herein. Therefore, the combination of these data sets allowed us to infer the directionality of the majority of the Hfq-enriched peaks. As a result, we were able to categorize most Hfq-enriched peaks into the following groups: (1) those associated with open reading frames (ORF), (2) those associated with 5' leader regions, (3) those corresponding to sRNAs, and (4) those corresponding to tRNA or rRNAs (Fig. 2C). Also, a small but notable class of Hfq-enriched peaks could not be easily placed into these categories (Table 2).

### mRNAs and mRNA Coding Region Fragments that Associate with Hfq

**Figure 3:**
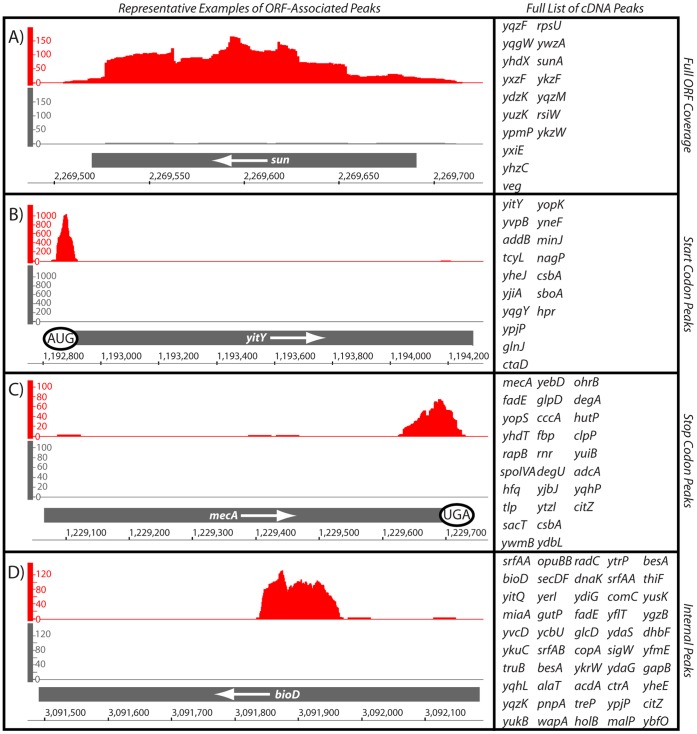
Representative Hfq-enriched peaks associated with open reading frames (ORF). (A) An individual representative example of a complete mRNA sequence that was enriched by coIP of Hfq is shown. The full list of genes sharing this pattern is shown to the right. (B) An individual example of an Hfq-enriched peak that overlaps with the translational start codon is shown. Other genes sharing this arrangement are listed to the right. (C) A representative example of an Hfq-enriched peak that overlaps with the translation stop codon is shown. Other genes sharing this arrangement are listed to the right. (D) An individual representative example of an Hfq-enriched peak located within an ORF is shown. Other genes sharing this arrangement are listed to the right. More details on these peaks are included in Table S2. The y-axis shows the volume of cDNA reads as a function of genomic position (x-axis) as determined by Illumina-based sequencing of Hfq-associated RNA molecules. Data shown in gray correspond to the negative control reaction (coimmunoprecipitation of Hfq that lacked an epitope tag).

The most abundant class of RNAs enriched by Hfq corresponded to the ORF portion of mRNAs (Table S2). Specifically, over 60% of the Hfq-enriched peaks mapped to either a portion of an ORF or encompassed an entire ORF. As such, we subdivided these peaks into four distinct categories based on the position of the mapped reads relative to the message: full ORF coverage, overlap with the start codon, overlap with the stop codon, and peaks that were internally located in ORFs (Fig. 2C; Fig. 3; Table S2). Hfq-enriched peaks fully encompassed 17 ORFs, which mainly corresponded to genes of unknown function (Fig. 3A). All of these ORFs are short and appear to encode small proteins. The longest mRNA to be enriched by Hfq was 276 nucleotides long and the shortest was 110 bases; the average length was 203 nucleotides (Table S2).

17 Hfq-enriched RNA fragments corresponded to a portion of the ORF that included the start codon (Fig. 3B; Table S2). These fragments ranged from 54 to 178 nucleotides in length (with a mean of 104). Many sRNAs affect expression of target mRNAs through base-pairing interactions with or near the ribosome-binding site [4]. It has also been demonstrated that sRNAs need not necessarily target ribosome binding site sequences directly in order to affect translation efficiency; instead, they just need to pair within the first five codons in order to reduce translation [48]. Therefore, this class of Hfq-bound RNAs is intriguing as it may conceivably correspond to sites of Hfq-mediated sRNA action in *B. subtilis*, although there are also other potential explanations.

Another group of Hfq-associated RNA molecules corresponded to peaks that overlapped with the stop codon (Fig. 3C; Table S2). In total 27 peaks fit this criterion, which ranged in size from 70 to 271 nucleotides in length. It is currently unclear what relationship, if any, may exist between Hfq and this region of mRNA ORFs. However, a potential interaction between Hfq and both start and stop codons has been postulated previously [49], which is potentially corroborated by our aggregate data. Interestingly, several Hfq-enriched peaks appeared to correspond to portions of mRNAs that included the 3′ UTR. However, since we could not unambiguously determine the directionality of these particular peaks, we placed them into a category with other intriguing but unknown RNA molecules (Table 2). Interestingly, a recent publication also demonstrated that many *Salmonella typhimurium* 3′ UTRs associate specifically with Hfq [50]. Many of these latter mRNA fragments were postulated to function as regulatory RNAs. It remains to be determined whether Hfq-associated 3′ UTRs might serve similar functions in *B. subtilis*. Finally, a large group consisted of enriched reads located within internal regions of ORFs. Approximately 45% of the ORF-associated peaks were placed into this category, ranging from 52 to 425 nucleotides in length (Fig. 3D). These Hfq-enriched RNA molecules may correspond to stable degradation intermediates, alternative ORFs, new sRNAs, sites of direct Hfq association, or pairing sites for Hfq-assisted sRNAs.

### Long mRNA Leader Regions Associate with Hfq

**Figure 4:**
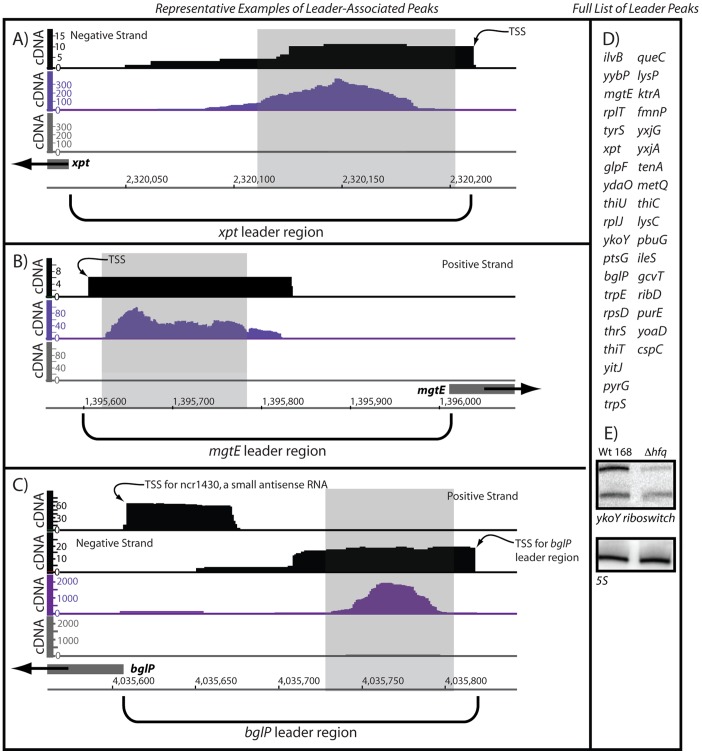
Hfq enrichment of signal-responsive, *cis*-acting regulatory RNAs. (A) An individual representative example is shown for Hfq-mediated enrichment of the leader region of a purine-sensing riboswitch [68]. The ligand-binding aptamer region is denoted by a shaded box to indicate the overlap between this sensory domain and the RNA sequences found to associate with Hfq. Previously measured transcription start site (TSS) mapping data (shown in black; [33]) is also shown in the figure as a means of denoting the 5′ end of the leader region. (B) An example is shown for Hfq-mediated enrichment of the leader region of a magnesium-sensing riboswitch [69]. The magnesium-sensing domain [70] is denoted by a shaded box. (C) Representative data are shown for Hfq-mediated enrichment of the leader region of a protein-responsive cis-acting regulatory RNA. Specifically, the *bglP* operon has been previously postulated to be subjected to multiple layers of regulatory control, including by transcription initiation factors, a protein-responsive leader region, and a small, putative antisense RNA that appears to base pair with the ribosome binding site. Although the latter antisense RNA was modestly enriched by coIP with Hfq, the protein-binding aptamer region was highly enriched by Hfq in this study (shown in purple). (D) Other leader regions that associated with Hfq are listed herein. More details on these RNAs are included in Table S3. Throughout the figure, the y-axis shows the volume of cDNA reads as a function of genomic position (x-axis) as determined by Illumina-based sequencing of Hfq-associated RNA molecules. Data shown in gray correspond to the negative control reaction (coimmunoprecipitation of Hfq that lacked an epitope tag). Data shown in black correspond to a prior transcription start site (TSS) mapping study and are included in this figure as a means of denoting the 5′ ends [33].

Another large class of Hfq-enriched signals that we found in our data corresponded to cis-acting, signal-responsive regulatory RNA elements, located within 5′ leader regions. Approximately 80 such signal-responsive RNA sequences have been previously identified within the *B. subtilis* genome [51]–[52]. Virtually all of these leader regions are greater than 100 nucleotides in length and are predicted to fold into complex secondary and tertiary structure arrangements. Tertiary folding of the sensory-responsive portion of these sequences (aptamer domain) typically correlates with association of the appropriate ligand. Binding of the ligand molecule(s) subsequently leads to repression or activation of downstream gene expression through modulation of transcription attenuation or the efficiency of translation initiation [53]. In *B. subtilis*, there are classes of cis-acting, signal-responsive regulatory RNAs that respond to different types of ligand molecules, including metabolites, metals, RNA-binding proteins, and selected tRNAs [51]–[52]. Overall, 19% of the Hfq-enriched peaks corresponded to previously characterized leader regions (Fig. 2C; Fig. 4). Interestingly, there did not appear to be a strong bias with respect to the classes of signal-responsive leader regions that were enriched by Hfq. In other words, Hfq-enriched peaks were observed for leader regions that responded to all types of signaling molecules including magnesium, metabolites, proteins, and tRNAs; however, not all members of a particular class were enriched by Hfq. For example, only four of the 19 known tRNA-sensing regulatory RNAs were enriched by Hfq.

One aspect of massively parallel sequencing technology that contrasts to most conventional RNA profiling methods is the ability to resolve data at individual nucleotide resolution. This level of resolution allowed for the examination of exactly which portions of signal-responsive leader regions co-immunoprecipitated with Hfq. Signal-responsive regulatory RNAs can be thought of as consisting of two portions: a ligand-sensing structural domain (aptamer) followed by downstream sequence elements that control gene expression in response to ligand-induced conformational changes. Interestingly, all of the Hfq-enriched peaks for signal-responsive leader regions corresponded almost exclusively to the aptamer domain (see Fig. 4 for representative examples). We hypothesize that the most likely explanation is that these structured regions are recalcitrant to digestion by cellular RNases, and therefore accrue within cells to the degree that they associate nonspecifically with Hfq hexamers. Alternatively, Hfq may associate with these leader regions because it has a yet-to-be determined role in their regulatory functions. As an initial test of the significance of this interaction, a putative Hfq-enriched putative aptamer (*ykoY*) was examined by northern blotting for wild-type and Δ*hfq* strains (Fig. 4E). Interestingly, the signal for this sequence was reduced in the Δ*hfq* strain, suggesting that Hfq may indeed affect its intracellular abundance. Nonetheless, it still remains to be determined whether the putative interaction between Hfq and mRNA leader regions is functionally meaningful.

In addition to associating with previously identified cis-acting regulatory sequences, Hfq also co-immunoprecipitated with 5′ leader regions that are likely to correspond to new regulatory elements. Specifically, several uncharacterized 5′ leader regions exhibited similar coIP patterns as with known riboswitches (Table 2). For example, the 5′ leader region for the *guaA* gene exhibited a coIP pattern that was essentially identical to several proximally located guanine-sensing riboswitches, suggesting that it might also correspond to a signal-responsive regulatory RNA. Indeed, manual inspection of the *guaA* leader region revealed the presence of a putative intrinsic transcription terminator site, consistent with a transcription attenuation-based regulatory element located within the 5′ leader region (Fig S3). Therefore, we speculate that the *guaA* leader region is likely to contain a newly identified but uncharacterized transcription attenuation system. *B. subtilis* already utilizes several transcription attenuation-based regulatory mechanisms for regulation of purine and pyrimidine levels [52], [54]. For example, UMP levels are sensed by an RNA-binding protein, PyrR, to control expression of pyrimidine biosynthesis genes via transcription attenuation. Also, the *pyrG* gene is regulated by a unique transcription attenuation mechanism, whereby conditions of low CTP stimulate reiterative addition of G residues, which then act to stabilize an antiterminator helix and promote downstream expression. Additionally, other *B. subtilis* purine biosynthesis genes are regulated by a total of five guanine-sensing and adenine-sensing riboswitches [52]. We therefore speculate that the *guaA* leader constitutes yet another post-initiation regulatory mechanism dedicated to nucleotide homeostasis. Unlike guanine- and adenine-sensing riboswitches, we did not observe any purine-induced changes in *guaA* secondary structure by preliminary structural probing assays (data not shown); therefore, the *guaA* leader region is likely to use a mechanism other than direct sensing of purine levels. In general, these data suggest that the putative relationship between Hfq and leader regions is close enough that Hfq coimmunoprecipitation profiles may assist discovery of new signal-responsive regulatory RNAs.

Signal-responsive cis-acting regulatory RNAs typically act through modulation of transcription elongation (attenuation) or by affecting the efficiency of translation initiation. Curiously, Gram-positive bacteria preferentially utilize transcription attenuation-based mechanisms while Gram-negative bacteria prefer to modulate translation initiation [51]–[52]. The molecular basis for this evolutionary preference is not yet known. However, one potential consequence could be that Gram-positive bacteria may accumulate many more prematurely terminated 5′ leader regions relative to Gram-negative bacteria, due to their greater reliance on transcription attenuation-based regulatory strategies. These 5′ leader regions usually do not encode for ORFs and are typically 150–400 nucleotides in length with an intrinsic terminator helix at their 3′ terminus. The overall arrangement of these RNAs qualitatively resembles that of sRNA regulatory molecules. Therefore, the observation of widespread interactions between Hfq hexamers and 5′ leader regions suggests that *B. subtilis* Hfq may face unique challenges in associating with specific sRNA molecules based upon potentially high interference by sRNA-like 5′ leader regions.

### sRNAs that Associate with Hfq

**Figure 5:**
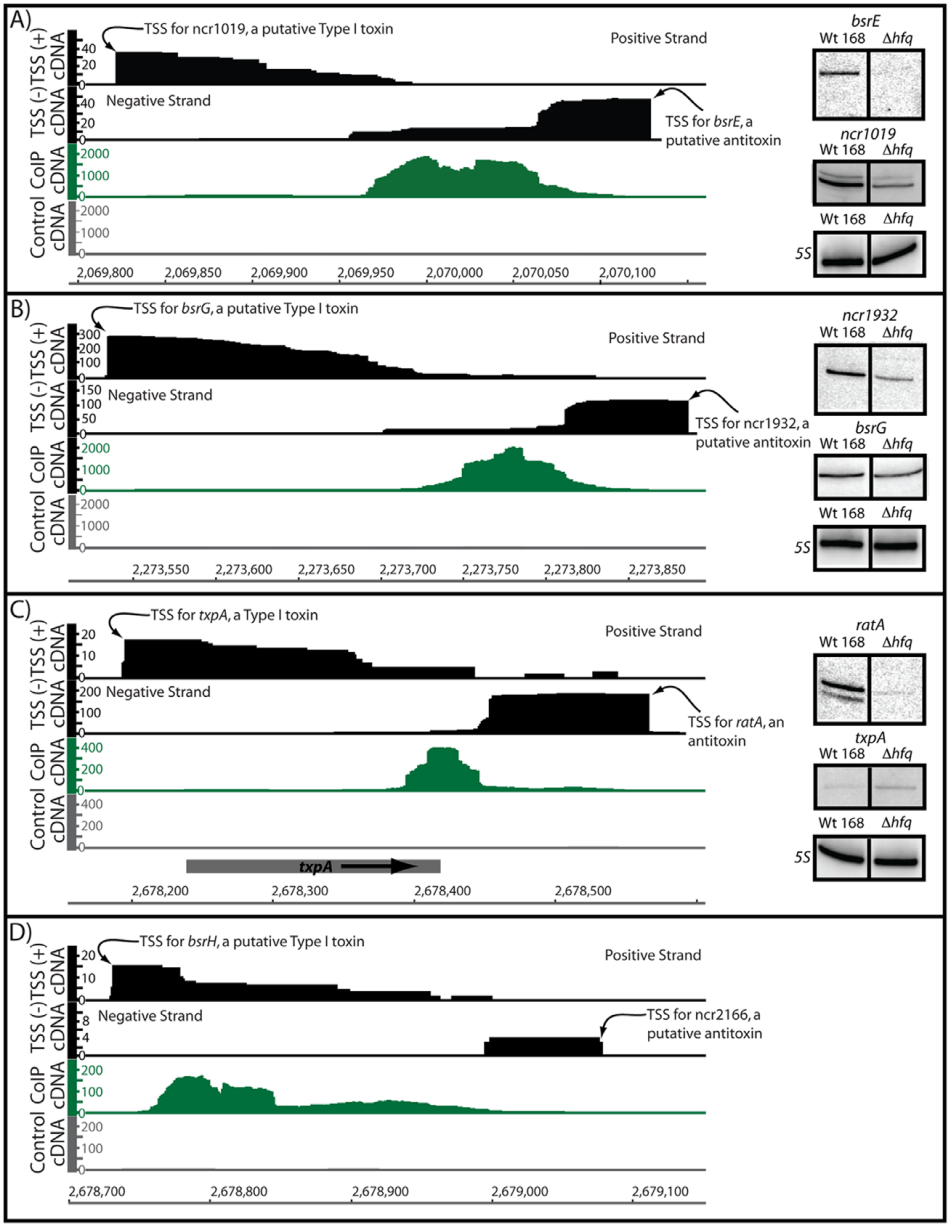
Hfq associates with type I antitoxin transcripts. (A–D) A previous study of *B. subtilis* transcription start sites (TSS) [33] revealed the presence of at least four putative type I toxin:antitoxin systems. The TSS data from this study are shown in black in this figure to indicate the orientation of these RNA transcripts, relative to regions of Hfq enrichment. In this study, Hfq appeared to preferentially associate with the antisense transcripts. In particular, the sequences that appeared to coIP with Hfq corresponded most often to the portions of the antitoxin transcripts that are predicted to base pair with the toxin-encoding mRNAs. Located to the right are northern blots for each corresponding toxin:antitoxin pair for wild-type and Δ*hfq* strains. For each set of northern blots the top panel corresponds to the anti-toxin, the middle panel the toxin, and the bottom panel is a 5S loading control. We were unable to detect *bsrH* or *ncr2166* transcripts by northern blotting. Throughout the figure, the y-axis shows the volume of cDNA reads as a function of genomic position (x-axis) as determined by Illumina-based sequencing of Hfq-associated RNA molecules. Data shown in gray correspond to the negative control reaction (coimmunoprecipitation of Hfq that lacked an epitope tag). Data shown in black correspond to a prior transcription start site (TSS) mapping study [33].

**Figure 6:**
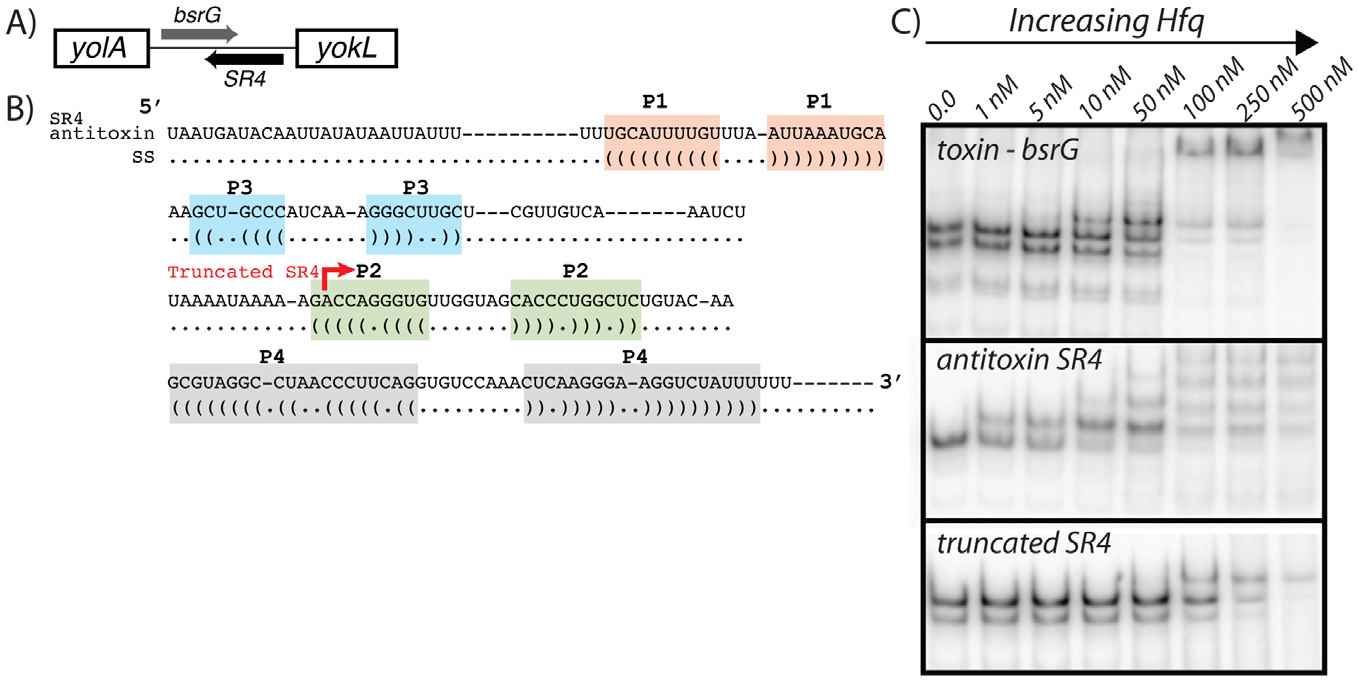
Hfq associates with type I toxin and antitoxin transcripts in vitro. (A) A schematic is shown for one of the four putative type I toxin:antitoxin systems that have been identified in *B. subtilis*. The primary sequence for the antitoxin is shown in (B), and colored regions denote portions of putative secondary structure. (C) Electrophoretic mobility shift assays showing association of purified hexahistidine-tagged Hfq with 5′ radiolabeled transcripts. For this experiment, the antitoxin sequence shown in (B) was transcribed in vitro by T7 RNA polymerase, except that three Gs were also added at the 5′ terminus to aid efficiency of transcription initiation. The 5′ terminus of the truncated antitoxin transcript is denoted by a red arrow. The radiolabeled transcripts were incubated with increasing concentrations of purified Hfq and resolved by nondenaturing polyacrylamide gel electrophoresis.

Over 100 putative sRNAs have been discovered in *B. subtilis* [27], [33], although only a few have been experimentally validated. Moreover, none of the *B. subtilis* sRNAs with proven mRNA targets have been found to be influenced by Hfq [24], [29], [36], [55]–[56]. Of the Hfq-enriched peaks identified in this study, approximately 11% corresponded to putative sRNAs (Fig. 2C; Table 1). One interesting class of sRNAs involved type I toxin:antitoxins. From prior studies, at least five type I toxin:antitoxin systems (type I TA) have been discovered in *B. subtilis* [33], [36]–[37]. These enigmatic systems are usually encoded on plasmids or are integrated into the host genome in prophage regions, where they are hypothesized to serve as “addiction” modules ensuring that the parasitic genetic element is retained in the genome. However, it has also been speculated that they may also function in other, unknown physiological functions [57]. For example, the BsrG/SR4 TA system in *B. subtilis* has recently been characterized as a temperature responsive TA module leading to cell lysis under heat shock conditions [58]. In general, type I TA systems consist of a stable toxin gene, which encodes for a small hydrophobic peptide capable of inserting into the plasma membrane, and an unstable anti-toxin which pairs through antisense interactions with the toxin mRNA. Interestingly, sRNAs corresponding to four of the five putative TA systems were enriched by Hfq in this study (Fig. 5). More specifically, the Hfq-enriched portion mostly corresponded with the sequences involved in antisense pairing between the toxin and antitoxin transcripts. Significantly more cDNA reads corresponded overall to the antisense transcript than with the sense toxin transcript. To determine whether Hfq would associate with these transcripts in vitro we purified hexahisitidine-tagged Hfq and monitored RNA-binding activity for the BsrG/SR4 toxin:antitoxin pair by electrophoretic mobility shift assays (Fig. 6). This experiment confirmed the interaction of these RNAs with Hfq. These data also revealed that the binding affinity of purified Hfq with SR4 antitoxin was decreased upon truncation of the 5′ terminus. This portion of SR4 is notably enriched in A/U residues, which may prove to be important for binding of *B. subtilis* Hfq.

Together, these observations suggest that the antisense sRNAs may be co-immunoprecipitated by Hfq. To test whether this interaction has consequences in vivo, the toxin and antitoxin transcripts were measured by northern blotting for wild-type and Δ*hfq* strains (Fig. 5). Deletion of Hfq resulted in a modest decrease in abundance for antitoxin RNAs by northern blotting, but had an even lesser effect on toxin mRNA levels. One might expect that decreased expression of an antitoxin might lead to decreased growth rate as a result of an increase in toxin production. Yet we did not observe an obvious growth rate defect for the Δ*hfq* strain. However, many prior genetic experiments that showed evidence of toxin activity (*i.e.,* toxin-mediated cellular death) were performed only under conditions where the toxin was artificially overexpressed. It is therefore possible that a phenotype from increased toxin production in a Δ*hfq* background only emerges under specific conditions where the toxin:antitoxins are most physiological relevant, which have yet to be identified.

Recently a bioinformatics approach was utilized to identify new type I TA systems in bacteria [37]. One new toxin candidate that was identified in *B. subtilis* was *yonT*, which is a gene of unknown function encoded within the SPβ prophage region of the *B. subtilis* 168 genome. The authors also detected an anti-sense transcript corresponding to a putative anti-toxin to *yonT*, which is also tentatively supported by our prior TSS mapping data (Fig. S4). Interestingly, an Hfq-enriched peak corresponded specifically to the 3′ region of *yonT*, in the region that would be expected to correspond to an antitoxin transcript, similar to the other four type I TA systems. We speculate from these aggregate data that Hfq may generally associate with anti-toxin transcripts, or, alternatively, with fragments of their degradation pathways. Similarly, we simply do not know why decreased abundance of RatA (or other antitoxins for that matter) doesn’t lead to decreased growth rate due to enhanced toxin production; however, we speculate that, again, unknown issues pertaining to the copy number of toxins and antitoxins might explain the apparent conflict. Interestingly, most genetic experiments that show evidence of toxin activity (i.e., toxin-mediated cellular death) only occur under conditions where the toxin was artificially overexpressed and not under conditions of “endogenous” expression levels. Therefore, the functions and expression patterns of toxin:antitoxin transcripts are somewhat enigmatic and for all of these reasons, we prefer to simply state our observation and allow for follow-up studies to discover the basis of the conundrum.

**Figure 7:**
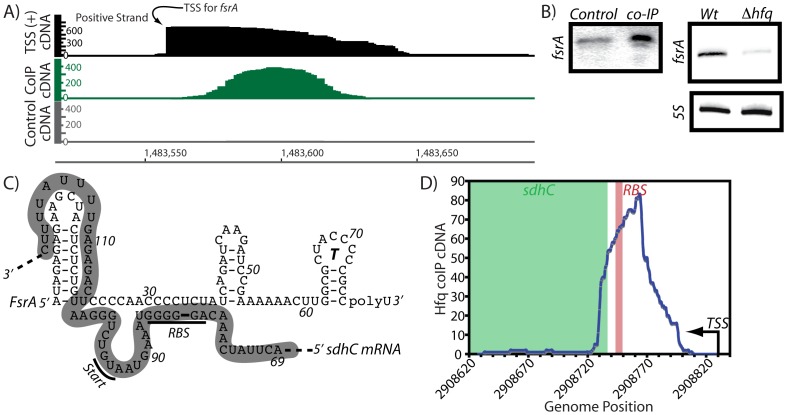
Hfq associates with the iron homeostasis sRNA regulator, FsrA. Previously, an sRNA regulator (FsrA) was identified as an important regulator of iron homeostasis genes [29]. The transcription start site of *fsrA* was identified previously [29], [33] and is shown in panel (A). In this study, Hfq is shown to coIP with the FsrA sRNA. In this panel, the y-axis shows the volume of cDNA reads as a function of genomic position (x-axis) as determined by Illumina-based sequencing of Hfq-associated RNA molecules. Data shown in gray correspond to the negative control reaction (coimmunoprecipitation of Hfq that lacked an epitope tag). Data shown in black correspond to a prior transcription start site (TSS) mapping study [33]. (B) Deletion of *hfq* resulted in moderately lowered abundance of FsrA, as ascertained by northern blotting analysis. (C) A well-characterized mRNA target of the FsrA sRNA is the ribosome binding site region of *sdhC* [29]. The predicted sRNA:mRNA pairing region is shown herein. The *sdhC* leader region is indicated in this figure by gray shading. (D) The region of the *sdhC* transcript that is predicted to pair with FsrA also appeared to coIP with Hfq in this study. The *sdhC* transcription start site is indicated with an arrow. The ribosome binding site is denoted by a red box and the *sdhC* coding region is shown in green. One speculative explanation for these data is that Hfq might assist FsrA:*sdhC* intermolecular interactions, although other explanations are still possible.

Among the putative trans-acting sRNAs that have been discovered in *B. subtilis* [27], [33], only a few of their corresponding mRNA targets have been identified. Among these are: (1) FsrA, which was identified as a Fur-regulated sRNA involved in the iron sparing response, (2) BsrF, which is activated by the global regulator CodY in response to branched chain amino acids and GTP levels, (3) SR1, which controls arginine catabolism, and (4) RsaE, which is a regulator of central metabolism genes that is widespread among diverse Gram-positive bacteria [25]–[26], [29], [39], [59]. In this study, FsrA was enriched (11-fold) upon coIP of Hfq, with cDNA reads fully encompassing the sRNA (Table 1; Fig. 7A). FsrA could also be easily detected by northern blot analysis of RNA molecules that coIP with Hfq (Fig. 7B), confirming the enrichment by Hfq. Interestingly, deletion of *hfq* resulted in a moderately decreased signal for FsrA, suggesting that there could be a relationship between Hfq and FsrA abundance In *B. subtilis*, FsrA functions to suppress enzymes that require iron as a cofactor by pairing with the ribosome binding sites for reduction of translation initiation efficiency [29]. One of these mRNA targets is the iron-containing enzyme, succinate dehydrogenase (*sdh*), which participates in the tricarboxylic acid cycle and is a common post-transcriptional regulatory target in other microorganisms. In this study, the *sdh* leader region was also identified as a putative ligand for association with Hfq, therefore, both an sRNA and its putative mRNA target locus were shown to associate with Hfq (Fig. 7D). It is therefore tempting to speculate that these Hfq-enriched sRNA and mRNA peaks specifically corresponded to processing of the RNAs after sRNA:mRNA base-pairing interactions. If so, it is possible that other ORF-associated Hfq-enriched peaks in this data set may also correspond to sites of sRNA action.

In total, only a small subset of sRNAs that have been identified previously co-immunoprecipitated with Hfq in this study (Table 1). Moreover, 32% of these Hfq-bound RNAs corresponded to antisense transcripts or type I antitoxins rather than “classical” sRNAs. It is also important to note that these Hfq-enriched RNAs did not simply correspond to those sRNAs that exhibited the highest expression levels ([33] and data not shown). In contrast, at least half of *Salmonella* sRNA candidates were co-immunoprecipitated by Hfq in a similar study [23], [60]. Therefore, unlike the gamma-proteobacterial species, *B. subtilis* Hfq appeared to associate broadly, yet selectively, with sRNA molecules. The molecular basis for recognition of this sRNA subset by Hfq still remains to be identified, as does its functional consequences. Preliminary searches were unable to identify a signature sequence unique to the subset enriched by Hfq.

**Figure 8:**
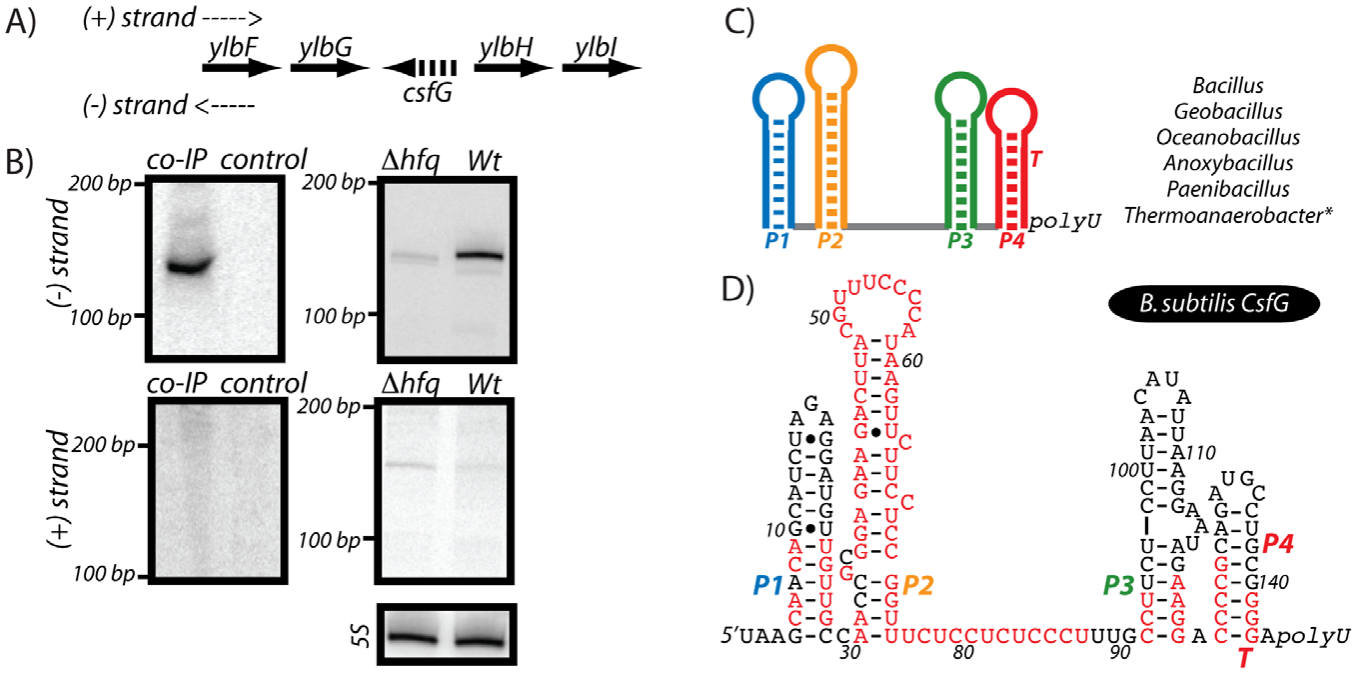
Coimmunoprecipitation of CsfG, an sRNA conserved in *Bacillaceae*, with Hfq. (A–B) Previously, an orphan riboswitch was identified upstream of the *ylbH* gene in certain *Bacillaceae* [61], [71]–[72]. In this study, we find that this RNA element is not likely to be conserved as a 5′ leader region for the *ylbH* gene, but is instead transcribed from the reverse genomic strand as a putative sRNA, which has been recently coined *csfG* [62]. This region was detected by northern blot analyses using oligonucleotide probes to hybridize to the region upstream of the *ylbH* gene (+ strand) and to the reverse complement of that sequence (−strand). A signal was only observed for an RNA sequence transcribed from the reverse complement strand, consistent with synthesis of CsfG. This putative sRNA also appeared to coIP with Hfq by northern blot analysis. Furthermore, the intracellular abundance of the sRNA was moderately decreased in a Δ*hfq* strain. (C–D) Consensus secondary structure diagram of the recently identified sRNA, CsfG (data not shown; [62]). Residues in red represent 95% conservation. This primary sequence and secondary structure consensus pattern was generated from covariance model searches (data not shown).

In addition to these Hfq-associated RNA molecules, several Hfq-enriched peaks did not correspond to previously identified sRNAs but still exhibited sRNA-like features (Table 1). We therefore designated these signals as putative sRNAs. One of these putative sRNAs is particularly noteworthy because the Hfq-enriched peak overlapped extensively with a portion of the genome that included an orphan riboswitch located upstream of an unknown gene, *ylbH* [61]. At first glance, it was tempting to categorize this Hfq-enriched peak as corresponding to the 5′ leader region of the *ylbH* gene; however, this RNA sequence could not be detected by northern blot analysis (Fig. 8). Instead, a strong signal was detected using an oligonucleotide probe that hybridized to the reverse complement sequence. This result is consistent with the recent identification of an sRNA encoded on the relevant genomic strand that was coined CsfG [62]. We therefore propose that the Hfq-associated RNA in this study corresponds to the CsfG sRNA. This particular sRNA exhibits a high degree of sequence conservation, particularly corresponding to CU-rich oligonucleotide stretches located within terminal loops or interhelical regions. To assess whether the interaction between CsfG and Hfq may be important in vivo, levels of CsfG were examined for wild-type and Δ*hfq* by Northen blot analysis (Fig. 8). The signal for CsfG was modestly reduced for the *hfq* mutant strain, suggesting that Hfq may affect the intracellular abundance of CsfG.

In general, the discovery of several new putative regulatory RNAs by coIP of Hfq, including new putative sRNAs and transcription attenuation systems, demonstrates a clear utility for examination of Hfq ligands in Gram-positive bacteria. Even though the full range of Hfq cellular function(s) remain to be identified in these bacteria, it can, at minimum, potentially be employed as a tool for discovery of new candidate regulatory RNA molecules.

## Concluding Remarks

The recent development of tools enabling global transcriptome analyses, coupled with a large number of fully sequenced eubacterial genomes has uncovered a vast role for post-transcriptional gene regulation in bacteria. These technologies have fueled the discovery of many RNA-based regulatory mechanisms, most notably riboswitches and sRNAs. However, identifying putative RNA regulators in bacteria has become much easier than identification of their targets and mechanisms of action. For instance, in *Bacillus subtilis*, over 100 putative sRNAs have now been reported [27], [33], yet defined targets and functional roles have only been defined for a few. Indeed, much more is known about the roles and mechanisms of sRNAs for post-transcriptional gene regulation in Gram-negative organisms. This is partly due to the close, interdependent relationship between sRNAs and Hfq that is exhibited by Gram-negative bacteria, where Hfq has been extensively investigated. Given that Hfq has been less studied overall in Gram-positive bacteria, and is not required thus far for sRNA-mediated regulation in certain bacteria, such as *Staphylococcus aureus*, we investigated in this study the possibility that *B. subtilis* Hfq associates with intracellular RNAs. At the onset of our experiments we expected to find one of three possibilities: (1) Hfq does not generally interact with cellular RNAs, and therefore might be involved in other, unknown cellular duties, (2) Hfq specifically associates with some but not all sRNAs, and (3) Hfq associates to the majority of sRNAs that have been identified. Our data support the second hypothesis – that Hfq associates with only a subset of the putative sRNAs that have been discovered. Moreover, the interaction between Hfq and sRNAs was strong enough under our conditions for discovery of new, putative sRNAs, including the widely conserved CsfG sRNA. Together, this important information will aid future characterization of post-transcriptional regulatory networks in *B. subtilis*. These data also provide direct evidence that *B. subtilis* Hfq indeed exhibits RNA-binding properties that are sufficient in theory for facilitating sRNA-mRNA interactions in vivo.

Intriguingly, Hfq also generally associated with the ligand-binding portions of the majority of cis-acting, signal-responsive regulatory RNAs in *B. subtilis*. This finding was unexpected, although *E. coli* Hfq has previously been found to interact with a few such RNA elements. In fact, the association of Hfq with the 5′ leader regions of signal-responsive RNAs was so consistent in this study that we predict our data is likely to include new signal-responsive RNA elements, such as the 5′ leader region of the *guaA* gene that we speculate to be a cis-acting regulatory RNA. However, the potential purpose, and consequences, of the interactions between Hfq and 5′ leader regions remains to be investigated. *B. subtilis* expresses over 80 known signal-responsive, cis-acting regulatory RNAs, and the majority of these RNA elements control gene expression through transcription attenuation [52]. Therefore, each of these RNA elements is likely to be highly expressed as an independent transcript under conditions that favor transcription termination rather than antitermination. In our study, we found that proportionally more signal-responsive 5′ leader regions appeared to coIP with Hfq than sRNAs. We speculate from this observation that Hfq may face a greater challenge in associating with sRNA regulators in *B. subtilis*, where sRNAs may broadly compete for Hfq access with 5′ leader regions, than in *E. coli*, where only a few signal-responsive, transcription attenuation systems are utilized. Together, our findings suggest a broader role than previously appreciated for the in vivo RNA-binding activity of *B. subtilis* Hfq, The catalog of Hfq-associated intracellular RNAs, as presented here, can now be explored for the functional significance of these individual interactions.

## Experimental Procedures

### Co-immunoprecipitation of Hfq

Five ml of glucose minimal media [33] with 50 µg/ml tryptophan was inoculated by a single colony of either wild-type 168 or MD145 from a freshly streaked plate and incubated without shaking overnight at 37°C. 50 ml minimal medium was inoculated with 0.5 ml of the overnight culture (OD_600_ ∼1.5) and incubated shaking at 37°C overnight (approximately 20 hours). These cells were then induced with isopropyl β-D-2-thiogalactopyranoside (IPTG) to a final concentration of 1 mM and incubated shaking for five hours at 37°C. The cultures were transferred to 50 mL conical tubes and centrifuged at 2,900×g for seven minutes; the resulting cell pellet was flash frozen in liquid nitrogen and stored at −80°C. Lysates were prepared using a method reported elsewhere [23]. Briefly, cell pellets were resuspended in lysis buffer (20 mM Tris pH 8.0, 150 mM KCl, 1 mM MgCl_2_, 1 mM DTT and 10 mg/ml lyzozyme) at placed on ice for ten minutes. The cell suspension was then flash frozen by liquid nitrogen and thawed for two minutes in a 55°C heat block. Approximately 400 µl of sterile glass beads were added and the suspension was subjected to five rounds of vortexing for 30 seconds followed by 30 seconds on ice (5×). The lysate was then centrifuged at 20,000×g for 30 minutes. The supernatant was then transferred to a fresh tube and incubated with 35 µl of anti-FLAG M2 monoclonal antibody (Sigma) for 30 minutes while rocking at 4°C. Subsequently, 75 µl of protein A sepharose (Sigma), pre-washed in 1 ml of lysis buffer lacking lyzozyme, was added to each tube and rocked for an additional 30 minutes at 4°C. Each sample was then centrifuged for two minutes at 500×g and washed 5× with 500 µl lysis buffer. The mixture was then resuspended in 500 µl wash buffer with 300 mM sodium acetate, mixed with 500 µl of phenol:chloroform:isoamyl alcohol (25∶24:1 pH 7.8) and centrifuged at 20,400×g for ten minutes. The aqueous phase was collected from each separation and placed in a new tube with 1.5 ml of 100% isopropyl alcohol and placed at −20°C overnight. To the organic phase (containing the beads), 1 ml of cold acetone was added for protein precipitation. The precipitated RNA was next centrifuged at 20,400×g for 30 minutes, the supernatant was discarded and the pellet was washed with 1 ml of 70% ethanol followed by centrifugation at 20,400×g for 10 minutes. The supernatant was carefully removed and the pellet was air dried for ten minutes at room temperature followed by re-suspension in 20 µl of sterile distilled water. Roughly 1 µg of RNA for the coIP and mock control samples was used for generation of Illumina-compatible cDNAs. The samples were prepared for sequencing by following the manufacturer’s instructions of the Illumina mRNA sequencing kit, except that the initial polyA-enrichment and final gel purification steps were omitted. Instead of the latter, the cDNA was purified using Qiagen PCR cleanup kit after adaptor ligation. The processed samples were sequenced using an Illumina Genome Analyzer (GAIIx) housed within the DNA Sequencing Core Facility at the UT Southwestern Medical Center. The resulting cDNA sequences were mapped onto the *B. subtilis* genome (NC_000964.3) using ‘Burrows-Wheeler Aligner’ (BWA) software [63]. Subsequent data processing were done using SAMtools [64] and custom-made Python scripts. Mapped reads were visualized using Integrated Genome Browser (IGB).

### SDS-PAGE and Western Blot Analysis

Aliquots of bacterial culture (5 ml) were harvested at various growth stages by centrifugation at 2,900 for five minutes followed by storage at −80°C. Total cell lysates were prepared by thawing the cells on ice for ten minutes followed by resuspension in 500 µl of lysis buffer (20 mM Tris-HCl pH 7.5, 100 mM KCl, 1 mM MgCl, 1 mM DTT, and Sigma Protease Cocktail (P 8849) diluted 1∶100). The samples were boiled for 20 minutes followed by centrifugation for ten minutes at 20,400×g with subsequent collection of the supernatant. Total protein concentration was determined by the Bradford method and 2 mg of each sample was mixed with 5 µl of 6× SDS-loading buffer in a total volume 25 µl. The samples were boiled an additional five minutes and resolved on a 10–20% SDS-PAGE gel followed by transfer to a nitrocellulose membrane. Each blot was probed with a 1∶1000 dilution of anti-FLAG M2 monoclonal antibody (Sigma 1804) overnight at 4°C in 5% (w/v) non-fat milk/TBST followed by a 1∶5,000 dilution of horseradish peroxidase-conjugated sheep-anti-mouse IgG (GE Healthcare NA931V) for 30 minutes at room temperature. The blots were subsequently developed with ECL development reagent for one minute (GE Healthcare RPN 2106V) and exposed to autoradiography film. After exposure each blot was stripped in mild stripping buffer (200 mM glycine, 0.1% SDS, 1.0% TWEEN 20 at a final pH of 2.2) at room temperature for ten minutes with gentle agitation, followed by two ten minute washes with PBS and two ten minute washes with TBST. Each blot was then blocked for 30 minutes at room temperature with 5% non-fat milk/TBST and incubated overnight 1∶2000 with rabbit serum raised against *B. subtilis* Sigma A (a generous gift from Dr. Masaya Fujita), followed by a 1∶5,000 dilution of horseradish peroxidase-conjugated donkey anti-rabbit IgG (GE Healthcare NA934V) and developed as described above.

### RNA Purification

Bacterial cells were centrifuged at 2,900×g for five minutes and pellets were stored at −80°C. The cells were thawed for ten minutes on ice followed by resuspension in 750 µl LETS buffer (0.1 M LiCl, 10 mM EDTA, 10 mM Tris-HCl pH 7.4 and 1% SDS) and disrupted by continuous vortexing with 400 µl of sterile glass beads for four minutes followed by incubation at 55°C for five minutes. This suspension was centrifuged for ten minutes at 20,400×g and the supernatant was collected and mixed with 1 mL of TRI reagent (Ambion AM9738) and incubated at room temperature for 5 minutes. Subsequently, 200 µl chloroform was added to each sample and vigorously mixed for 15 seconds followed by incubation at room temperature for three minutes. The samples were centrifuged at 20,400×g for 15 minutes and the top 600 µl of the phase-separated mixture was collected and precipitated with 1 ml of isopropyl alcohol overnight at −80°C. Precipitated RNA was then pelleted and the supernatant was discarded. The pellet was washed with 200 µl of 70% ethanol, air-dried for 10 minutes at room temperature and resuspended in 50 µl purified water, followed by incubation at 55°C for five minutes.

### Quantitative Real-time RT-PCR

Total RNA (4 µg) was incubated with 1 µl of RQ1 RNase-free DNase (Promega MG10A) in 0.5 mM MgCl_2_ at a total volume of 20 µl for 30 minutes at 37°C followed by 10 minutes at 75°C for enzymatic inactivation. 1 µg of this RNA was converted to cDNA using the iScript cDNA Synthesis Kit (Bio-Rad 170–8891) per the manufacturer’s instructions. Quantitative PCR amplification was then performed using specific primer pairs (1.25 µM) and the appropriate template nucleic acids in the presence of iTaq SYBR Green with ROX (Bio-Rad 172–5850) on an ABI 7900 HT Fast Real Time PCR System. Subsequent data analysis was executed using the ABI SDS 2.2.2 software package. The primer pairs used were as follows MD399/400 (*hfq*) and MD421/422 (*sigA*).

### Northern Blot Analyses

Total RNA samples (15–20 µg) were heated at 65°C for ten minutes in gel loading buffer (45 mM Tris–borate, 4 M urea, 10% sucrose [w/v], 5 mM EDTA, 0.05% SDS, 0.025% xylene cyanol FF, 0.025% bromophenol blue) and resolved by 6% denaturing (8 M urea) polyacrylamide electrophoresis. RNAs were transferred to BrightStar-Plus nylon membranes (Ambion) using a semi-dry electroblotting apparatus (Owl Scientific) according to manufacturer instructions. The blots were UV-crosslinked and hybridized overnight at 42°C in UltraHyb-Oligo buffer (Ambion) with the appropriate 5′-radiolabeled (^32^P) DNA oligonucleotide (Table S4). The blots were then washed twice for 15 min using low stringency wash buffer (1×SSC, 0.1% SDS, 1 mM EDTA). Radioactive bands were visualized using ImageQuant or ImageJ software and a Typhoon PhosphorImager (Molecular Dynamics).

### Strain Construction

All strains were transformed with 1–10 µg plasmid DNA using a method described previously [65].

#### In-frame deletion: *Δhfq*

To generate the marker-less Δ*hfq* deletion construct, approximately 400 bp upstream of *hfq* gene, including the first codon, was amplified from *B. subtilis* 168 chromosomal DNA using primer pairs MD324/325 (Table S4). These oligonucleotides were engineered to contain an in-frame stop codon following the ATG start codon. They also introduced a *Not I* restriction site into the *hfq* gene. The second half of the construct was generated via PCR amplification using primer pairs MD326/MD327. This forward oligonucleotide (MD327) begins at the ATG of *hfq* and is directly complimentary to MD325, while the reverse oligonucleotide MD327 hybridizes with sequences located approximately 100 bases downstream of the *hfq* coding sequence. The two PCR products were mixed in equal amounts and sewn together through amplification with primers MD324/327; the resulting PCR product was then checked for digestion with *Not I*. This DNA was then sub-cloned into pMAD [66] via *EcoR1* and *BamHI* sites, which were added by the oligonucleotide primers and the correct clones were confirmed by DNA sequencing. The pMAD plasmid carries a temperature-sensitive origin of replication, an erythromycin resistance cassette and a constitutively active *lacZ* gene. Correspondingly, the pMAD-based plasmids were transformed into *B. subtilis* strain 168 at the permissive temperature for plasmid replication (30°C) with selection for resistance to erythromycin (1 µg ml^−1^) and lincomycin (25 µg ml^−1^) on plates containing bromo-chloro-indolyl-galactopyranoside (X-gal). To stimulate integration of the plasmids via Campbell recombination the cells were cultured overnight in 2xYT broth at the restrictive temperature (37°C) and then incubated on solid medium at 37°C with selection for resistance to erythromycin and lincomycin. The resulting isolates were blue from pMAD-encoded *lacZ* expression. To screen for recombination-based loss of the integrated plasmids, the strains were incubated overnight in 2xYT broth without shaking and without antibiotics at 30°C, followed by shaking incubation for 5 h at 30°C and then 3 h at 37°C. The cells were then serially diluted and plated on tryptose blood agar base (TBAB) at 37°C in the absence of antibiotic. Individual colonies were patched onto TBAB plates with and without erythromycin and lincomycin. Isolates that were sensitive to antibiotics, and were white on X gal-containing medium, were presumed to result from recombination-loss of the integrated plasmid. Chromosomal DNA was isolated from these strains and used as templates for diagnostic PCR reactions and subsequent DNA sequencing reactions to confirm mutagenesis of the targeted genomic locus.

#### Construction of an ectopic, inducible, Epitope-Tagged *hfq* allele

The *hfq* coding sequence was amplified using primers MD381/382 and sub-cloned via *HindIII* and *NheI* into pHyper-SPANK (a gift from David Rudner), an IPTG-inducible expression vector that integrates into the *amyE* gene. To ensure efficient translation the forward oligo used to amplify *hfq* added the ribosome binding site from the *rpsD* gene. Generation of a DNA template encoding the FLAG peptide sequence was accomplished via primer extension of overlapping oligonucleotides MD383/384, which regenerated the 3× FLAG repeat as encoded by the yeast vector p417-CYC-NTAP (a gift from Benjamin Tu, Sunil Laxman). This DNA fragment was then subcloned into pHyper-SPANK and fused to the 3′ terminus of the *hfq* coding sequence, resulting in plasmid pMD-145. Transformants were screened by diagnostic PCR, verified by DNA sequencing, and used for the co-immunoprecipitation experiments described herein.

#### Construction of a strain expressing an Epitope-Tagged *hfq* allele

The *hfq* gene containing a C-terminal 3× FLAG tag was amplified from pMD -145 using the oligonucleotide primers MD395/396 and sub-cloned into the pMAD vector via *BglII* and *EcoRI*. In order to preserve the C-terminal FLAG sequence during allelic exchange, 400 bp immediately downstream of the *hfq* coding sequence was amplified using MD397/MD398 and also sub-cloned into the above pMAD vector, downstream of the epitope-tagged copy of *hfq.* Positive clones were subsequently screened by diagnostic PCR and confirmed by sequencing, resulting in plasmid pMD-131. Transformation and allelic exchange using the pMAD-based plasmids are described above.

#### Electrophoretic mobility shift assays using purified Hfq

Hexahistidine-tagged *B. subtilis* Hfq, which was subcloned into the pHisII vector and transformed into *E. coli* BL21 (DE3) cells, was expressed and purified using a method published for *E. coli* Hfq [67]. Synthetic oligonucleotides (IDT) were used for PCR amplification of double-stranded DNA molecules that could be used as templates for transcription in vitro. Specifically, a promoter sequence for T7 RNA polymerase (RNAP) was added upstream of the BsrG toxin and SR4 antitoxin genes, so that these RNAs could be transcribed by T7 RNAP in vitro. The sequences of these RNAs are shown in Figure 6. The resulting transcripts were resolved by denaturing polyacryamide gel electrophoresis (PAGE), purified by UV shadowing, and radiolabeled with γ-^32^P ATP. RNA (∼2 fmol) was incubated with increasing concentrations of purified Hfq protein in a 10 µl reaction containing 50 mM Hepes pH 7.5, 150 mM sodium chloride, 10 mM MgCl_2_ and 2.5 ng/µl yeast tRNA for 30 minutes at 25°C. These samples were then resolved on non-denaturing 5% TBE polyacrylamide gels (5% acrylamide: bis (80∶1)). Gels were pre-run for 30 minutes at 40 volts, then electrophoresed at 40 volts for ∼2.5 hours with 0.5× TBE running buffer and dried for 45 mins. Gels were exposed to phosphor screens overnight and visualized using a PhosphorImager (GE Health Sciences).

## Supporting Information

Figure S1 **Expression of** ***hfq*** **.** (A) The Hfq sequences from *B. subtilis* and *E. coli* are shown as an alignment to highlight the C-terminal truncation in *B. subtilis* (and many other Gram-positive bacteria). (B) Approximately 600 *B. subtilis* transcription start sites were determined in a previous study [33]. We examined these data and found that they supported a single transcription start site upstream of *hfq*, suggesting that it is a monocistronic transcript.
(PDF)

Figure S2 **Co-immunoprecipitation of cellular RNAs that associate with** ***Bacillus subtilis*** **Hfq.** Hfq^FLAG^ was co-immunoprecipitated from cellular extracts of stationary phase cells using an α-FLAG monoclonal antibody. A cDNA library was then created from the Hfq-associated RNA molecules, which was subjected to high-throughput sequencing using an Illumina Genome Analyzer (see Experimental Procedures for more details).
(PDF)

Figure S3 **Discovery of a putative cis-acting transcription attenuation system upstream of** ***guaA*** **.** (A) Hfq coimmunoprecipitation revealed a coIP peak upstream of the *guaA* gene. Our prior transcription start site mapping data [33] revealed that a long leader region is situated upstream of *guaA*, consistent with earlier experimental evidence [76]. This leader region essentially encompasses the Hfq coIP peak. This pattern is consistent with the many signal-responsive leader regions that were found to coIP with Hfq in this study (see Figure 4 and Table S3 for more details). (B) Inspection of the *guaA* leader region revealed the presence of several putative secondary structural elements including a putative intrinsic transcription termination site. Most cis-acting regulatory RNAs in *B. subtilis* control gene expression by modulating transcription attenuation within a 5′ leader region. Therefore, we speculate that the presence of a premature termination site upstream of the *guaA* coding region is consistent with a transcription attenuation system, although experimentation will be required to test this hypothesis.
(PDF)

Figure S4 **Expression of a possible antitoxin for the** ***yonT*** **type I toxin.** Recently, several putative type I toxins were identified in the *B. subtilis* genome [37], including the *yonT* gene. Our analysis of the Hfq coIP data revealed that four examples of previously identified type I antitoxins appeared to exhibit preferential enrichment by Hfq. Inspection of the putative *yonT* toxin gene revealed an Hfq-associated peak located in the region where an antitoxin transcript would be most likely to occur. Therefore, we speculate that the Hfq-associated peak that overlaps *yonT* might correspond to an antitoxin transcript.
(PDF)

Table S1 **Hfq coIP peaks corresponding to tRNAs or with an RPKM ratio less than two.** The expression of each peak (described in the main text) was quantified in reads per kilobase per million mapped reads, or ‘RPKM’ [47]. The ratio of these values for the Hfq^FLAG^ and mock control samples was taken as an indicator of Hfq-mediated enrichment. Included in this table are the data corresponding to peaks that exhibited an RPKM ratio less than two, and the peaks corresponding to tRNA genes.
(PDF)

Table S2 **Hfq coIP peaks associated with mRNA coding regions.** The expression of each peak (described in the main text) was quantified in reads per kilobase per million mapped reads, or ‘RPKM’ [47]. The ratio of these values for the Hfq^FLAG^ and mock control samples was taken as an indicator of Hfq-mediated enrichment. Included in this table are data corresponding to peaks that are associated with different regions of mRNA coding regions.
(PDF)

Table S3 **Hfq coIP peaks associated with mRNA leader regions.** The expression of each peak (described in the main text) was quantified in reads per kilobase per million mapped reads, or ‘RPKM’ [47]. The ratio of these values for the Hfq^FLAG^ and mock control samples was taken as an indicator of Hfq-mediated enrichment. Included in this table are data corresponding to peaks that are associated with 5' leader regions.
(PDF)

Table S4 **Oligonucleotides used in this study.** Included in this table are the sequences and brief descriptions of the DNA oligonucleotides used in the experiments described by this manuscript.
(PDF)
