# Supplementary material for: Association of RNAs with Bacillus subtilis Hfq
Source: PLoS One. 2013 Feb 15;8(2):e55156. doi: 10.1371/journal.pone.0055156 (PMC3574147; doi:10.1371/journal.pone.0055156)
Supplement: Figure S1 — Expression of hfq . (A) The Hfq sequences from B. subtilis and E. coli are shown as an alignment to highlight the C-terminal truncation in B. subtilis (and many other Gram-positive bacteria). (B) Approximately 600 B. subtilis transcription start sites were determined in a previous study [33]. We examined these data and found that they supported a single transcription start site upstream of hfq, suggesting that it is a monocistronic transcript. (PDF) [file pone.0055156.s001.pdf]

```
A B. subtilis hfq MAKGQSLQDPFLNALRRERVPVSIYLVNGIKLQGQIESFDQFVILLKN-TVSQMVYKHAI 59
     E. coli hfq M-KPINIQDQFLNQIRKENTYVTVFLLNGFQLRGQVKGFDNFTVLLESEGKQQLIYKHAI 59
                   *  .:** *** :*:*.. *:::*:**::*:**::.**:*.:**:.   .*::*****

  B. subtilis hfq STFAPQK--------------------------NVQLELE---
     E. coli hfq STVVPSRPVSHHSNNAGGGTSSNYHHGSSAQNTSAQQDSEETE
                 **..*.:                          ..* : *
```

B

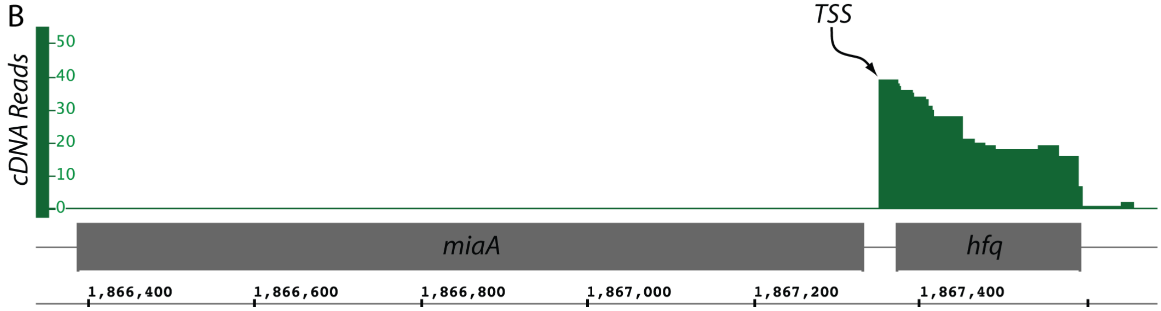

Figure S1. Organization of *hfq*. (A) The Hfq sequences from *B. subtilis* and *E. coli* are shown as an alignment to highlight the C-terminal truncation in *B. subtilis* (and many other Gram-positive bacteria). (B) Approximately 600 *B. subtilis* transcription start sites were determined in a previous study (Irnov *et al*., 2010). We examined these data and found that they supported a single transcription start site upstream of *hfq*, suggesting that it is a monocistronic transcript.
