# Supplementary material for: Association of RNAs with Bacillus subtilis Hfq
Source: PLoS One. 2013 Feb 15;8(2):e55156. doi: 10.1371/journal.pone.0055156 (PMC3574147; doi:10.1371/journal.pone.0055156)
Supplement: Figure S2 — Co-immunoprecipitation of cellular RNAs that associate with Bacillus subtilis Hfq. HfqFLAG was co-immunoprecipitated from cellular extracts of stationary phase cells using an α-FLAG monoclonal antibody. A cDNA library was then created from the Hfq-associated RNA molecules, which was subjected to high-throughput sequencing using an Illumina Genome Analyzer (see Experimental Procedures for more details). (PDF) [file pone.0055156.s002.pdf]

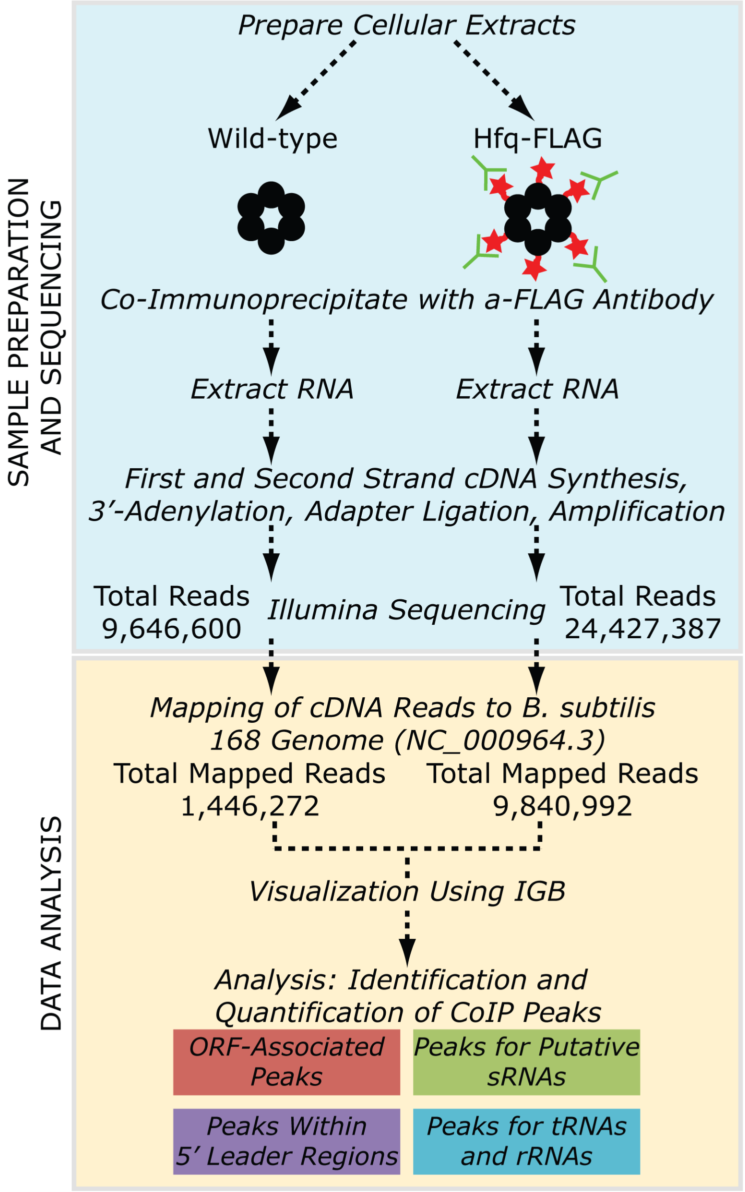

Figure S2. Co-immunoprecipitation of cellular RNAs that associate with *Bacillus subtilis* Hfq. Hfq^{FLAG} was co-immunoprecipitated from cellular extracts of stationary phase cells using an α-FLAG monoclonal antibody. A cDNA library was then created from the Hfq-associated RNA molecules, which was subjected to high-throughput sequencing using an Illumina Genome Analyzer (see Experimental Procedures for more details).
