# Supplementary material for: Association of RNAs with Bacillus subtilis Hfq
Source: PLoS One. 2013 Feb 15;8(2):e55156. doi: 10.1371/journal.pone.0055156 (PMC3574147; doi:10.1371/journal.pone.0055156)
Supplement: Figure S3 — Discovery of a putative cis-acting transcription attenuation system upstream of guaA . (A) Hfq coimmunoprecipitation revealed a coIP peak upstream of the guaA gene. Our prior transcription start site mapping data [33] revealed that a long leader region is situated upstream of guaA, consistent with earlier experimental evidence [76]. This leader region essentially encompasses the Hfq coIP peak. This pattern is consistent with the many signal-responsive leader regions that were found to coIP with Hfq in this study (see Figure 4 and Table S3 for more details). (B) Inspection of the guaA leader region revealed the presence of several putative secondary structural elements including a putative intrinsic transcription termination site. Most cis-acting regulatory RNAs in B. subtilis control gene expression by modulating transcription attenuation within a 5′ leader region. Therefore, we speculate that the presence of a premature termination site upstream of the guaA coding region is consistent with a transcription attenuation system, although experimentation will be required to test this hypothesis. (PDF) [file pone.0055156.s003.pdf]

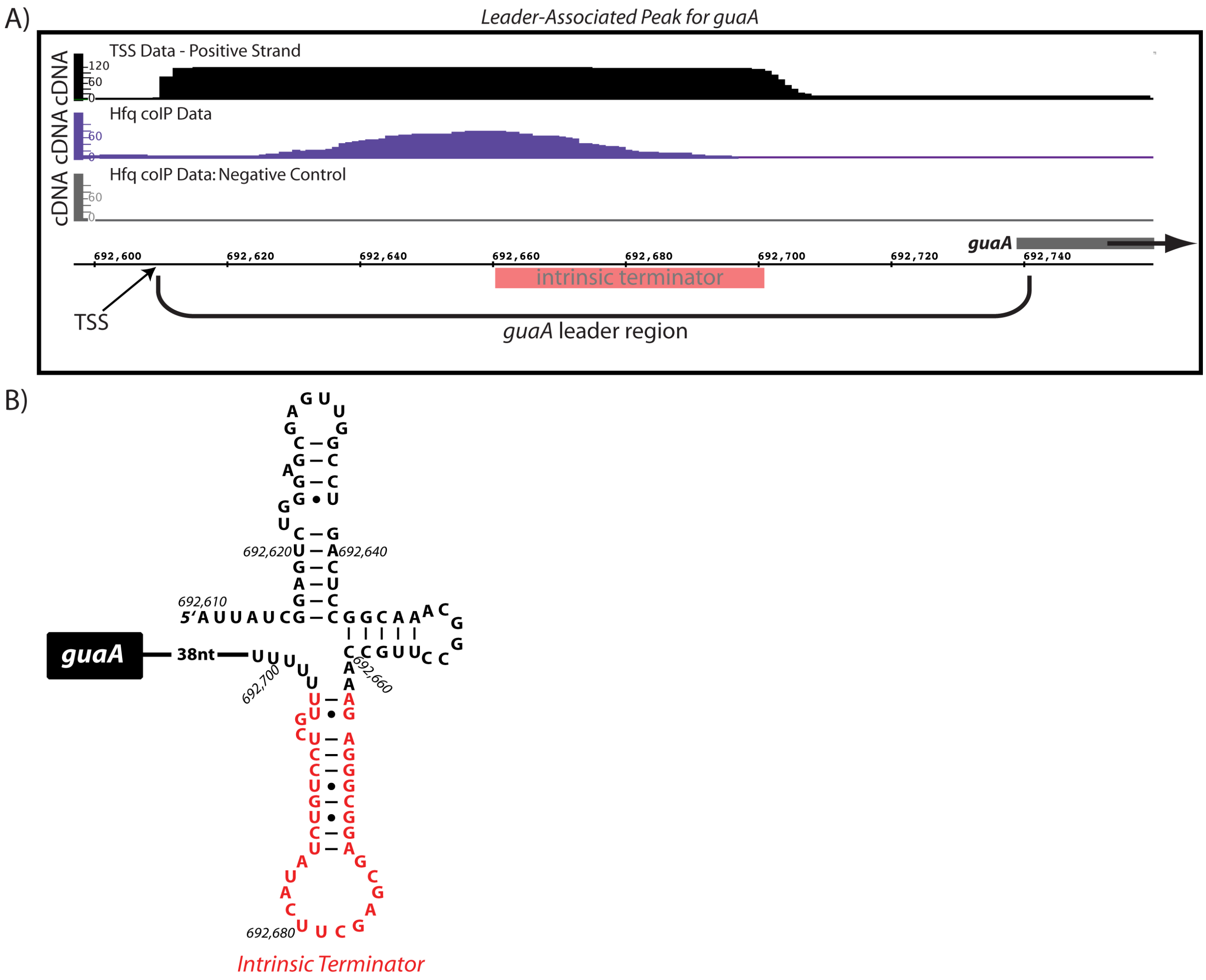

Figure S3. Discovery of a putative cis-acting transcription attenuation system upstream of *guaA*. (A) Hfq coimmunoprecipitation revealed a coIP peak upstream of the *guaA* gene. Our prior transcription start site mapping data (Irnov *et al*., 2010) revealed that a long leader region is situated upstream of *guaA*, consistent with earlier experimental evidence (Mäntsälä and Zalkin, 1992). This leader region essentially encompasses the Hfq coIP peak. This pattern is consistent with the many signal-responsive leader regions that were found to coIP with Hfq in this study (see Figure 4 and Table S3 for more details). (B) Inspection of the *guaA* leader region revealed the presence of several putative secondary structural elements including a putative intrinsic transcription termination site. Most cis-acting regulatory RNAs in *B. subtilis* control gene expression by modulating transcription attenuation within a 5’ leader region. Therefore, we speculate that the presence of a premature termination site upstream of the *guaA* coding region is consistent with a transcription attenuation system, although experimentation will be required to test this hypothesis.
