# Supplementary material for: Association of RNAs with Bacillus subtilis Hfq
Source: PLoS One. 2013 Feb 15;8(2):e55156. doi: 10.1371/journal.pone.0055156 (PMC3574147; doi:10.1371/journal.pone.0055156)
Supplement: Figure S4 — Expression of a possible antitoxin for the yonT type I toxin. Recently, several putative type I toxins were identified in the B. subtilis genome [37], including the yonT gene. Our analysis of the Hfq coIP data revealed that four examples of previously identified type I antitoxins appeared to exhibit preferential enrichment by Hfq. Inspection of the putative yonT toxin gene revealed an Hfq-associated peak located in the region where an antitoxin transcript would be most likely to occur. Therefore, we speculate that the Hfq-associated peak that overlaps yonT might correspond to an antitoxin transcript. (PDF) [file pone.0055156.s004.pdf]

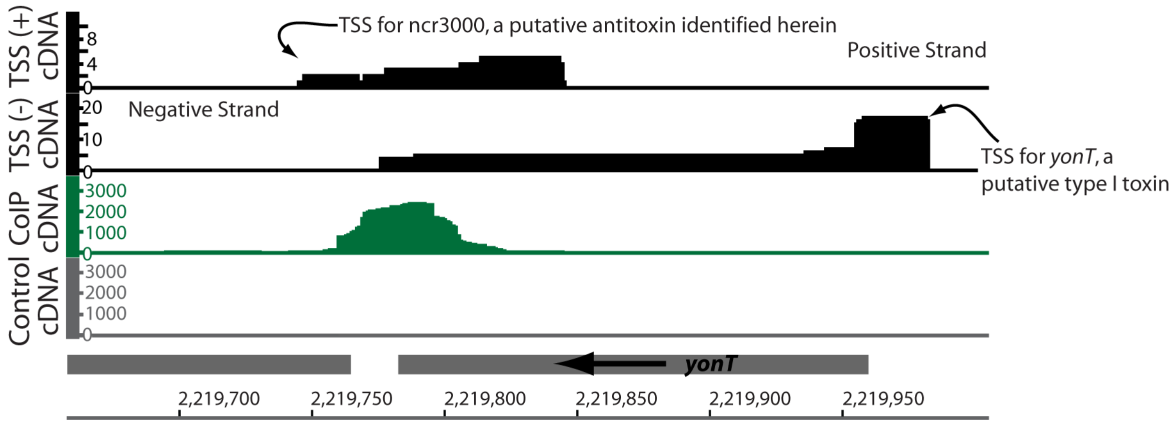

Figure S4. Expression of a possible antitoxin for the *yonT* type I toxin. Recently, several putative type I toxins were identified in the *B. subtilis* genome (Fozo *et al*., 2010), including the *yonT* gene. Our analysis of the Hfq coIP data revealed that four examples of previously identified type I antitoxins appeared to exhibit preferential enrichment by Hfq. Inspection of the putative *yonT* toxin gene revealed an Hfq-associated peak located in the region where an antitoxin transcript would be most likely to occur. Therefore, we speculate that the Hfq-associated peak that overlaps *yonT* might correspond to an antitoxin transcript.
