# Supplementary material for: Association of RNAs with Bacillus subtilis Hfq
Source: PLoS One. 2013 Feb 15;8(2):e55156. doi: 10.1371/journal.pone.0055156 (PMC3574147; doi:10.1371/journal.pone.0055156)
Supplement: Table S1 — Hfq coIP peaks corresponding to tRNAs or with an RPKM ratio less than two. The expression of each peak (described in the main text) was quantified in reads per kilobase per million mapped reads, or ‘RPKM’ [47]. The ratio of these values for the HfqFLAG and mock control samples was taken as an indicator of Hfq-mediated enrichment. Included in this table are the data corresponding to peaks that exhibited an RPKM ratio less than two, and the peaks corresponding to tRNA genes. (PDF) [file pone.0055156.s005.pdf]

Table S1 tRNA peaks and Hfq coIP peaks with an RPKM ratio of <2

Hfq coIP peaks with an RPKM ratio of <2				
**Gene**	**Peak Length**	**Coordinates**	**Coordinates**	**RPKM Ratio[a]**
trnQ-Arg	97	3545879	3545975	2.00
trnSL-Ser1	164	22289	22452	1.89
trnS-Leu2	123	529311	529433	1.78
trnD-Asp	254	952036	952289	1.77
trnD-Phe	254	952036	952289	1.77
trnS-Asn	570	528691	529260	1.74
trnS-Glu	570	528691	529260	1.74
trnS-Gln	570	528691	529260	1.74
trnS-Lys	570	528691	529260	1.74
trnB-Thr	98	3173596	3173693	1.68
trnSL-Met1	187	70168	70354	1.56
trnD-Gly	289	952699	952987	1.56
trnD-Leu1	289	952699	952987	1.56
trnD-Glu	251	951782	952032	1.54
trnD-Val	251	951782	952032	1.54
trnJ-Gly	455	95772	96226	1.51
trnJ-Arg	455	95772	96226	1.51
trnJ-Pro	455	95772	96226	1.51
trnJ-Ala	455	95772	96226	1.51
trnI-Arg	273	166061	166333	1.49
trnI-Pro	273	166061	166333	1.49
trnI-Ala	273	166061	166333	1.49
trnB-Met3	793	3172791	3173583	1.48
trnB-Ala	793	3172791	3173583	1.48
trnB-Arg	793	3172791	3173583	1.48
trnB-Leu2	793	3172791	3173583	1.48
trnB-Gly1	793	3172791	3173583	1.48
trnB-Leu1	793	3172791	3173583	1.48
trnSL-Ala1	91	3194446	3194536	1.48
yxzE	85	3983070	3983154	1.47
trnSL-Glu2	466	194160	194625	1.43
trnSL-Tyr1	466	194160	194625	1.43
trnA-Ile	188	31915	32102	1.39
trnA-Ala	188	31915	32102	1.39
trnD-Tyr	178	952299	952476	1.37
trnB-Phe	366	3172424	3172789	1.37
trnB-Asp	366	3172424	3172789	1.37
trnB-Met2	366	3172424	3172789	1.37
trnB-Ser1	366	3172424	3172789	1.37
trnD-Leu2	109	953192	953300	1.34
trnO-Ile	179	11455	11633	1.34
rrnI-16S	1559	160888	162446	1.33
rrnJ-16S	1592	90500	92091	1.32
rrnH-16S	1593	166461	168053	1.32
rrnA-16S	1559	30275	31833	1.32

rrnW-16S	1560	96388	97947	1.31
rrnD-16S	1563	946690	948252	1.31
component	327	26391	26717	1.31
rrnO-16S	1564	9803	11366	1.30
trnB-Ile2	273	3172150	3172422	1.30
trnB-Gly2	273	3172150	3172422	1.30
trnB-His	273	3172150	3172422	1.30
rrnE-16S	1569	635421	636989	1.30
rrnG-16S	1593	171459	173051	1.27
rrnB-16S	1569	3177084	3178652	1.27
trnD-His	174	952484	952657	1.25
trnD-Gln	174	952484	952657	1.25
trnSL-Arg2	108	2899808	2899915	1.24
trnSL-Gly1	93	967051	967143	1.23
trnI-Asn	152	165754	165905	1.18
trnB-Asn	274	3171873	3172146	1.18
trnB-Glu	274	3171873	3172146	1.18
trnE-Arg	170	635109	635278	1.13
trnE-Gly	170	635109	635278	1.13
trnSL-Val2	115	1262757	1262871	1.10
rrnJ-5S	507	95232	95738	1.03
trnJ-Val	507	95232	95738	1.03
SAM	200	1424502	1424701	0.96
trnD-Asn	309	951451	951759	0.92
rrnD-5S	309	951451	951759	0.92
rrnD-5S	309	951451	951759	0.92
trnB-Val	231	3173710	3173940	0.88
rrnB-5S	231	3173710	3173940	0.88
trnB-Val	231	3173710	3173940	0.88
rrnB-5S	231	3173710	3173940	0.88
trnSL-Arg1	78	2003272	2003349	0.86
rrnE-5S	220	640132	640351	0.83
trnE-Met	220	640132	640351	0.83
rrnE-5S	220	640132	640351	0.83
trnE-Met	220	640132	640351	0.83
rrnO-5S	140	14673	14812	0.71
rrnO-5S	140	14673	14812	0.71
rrnH-5S	136	171180	171315	0.71
rrnH-5S	136	171180	171315	0.71
rrnA-5S	133	35224	35356	0.71
rrnA-5S	133	35224	35356	0.71
rrnI-5S	127	165585	165711	0.71
rrnI-5S	127	165585	165711	0.71
rrnW-5S	136	101077	101212	0.71
rrnW-5S	136	101077	101212	0.71
rrnG-5S	127	176190	176316	0.70
rrnG-5S	127	176190	176316	0.70
rrnI-23S	2925	162610	165534	0.56
rrnE-23S	2945	637138	640082	0.56

rrnE-23S	2945	637138	640082	0.56
rrnB-23S	2914	3173995	3176908	0.56
rrnB-23S	2914	3173995	3176908	0.56
rrnJ-23S	2935	92249	95183	0.56
rrnJ-23S	2935	92249	95183	0.56
rrnW-23S	2915	98120	101034	0.56
rrnW-23S	2915	98120	101034	0.56
rrnO-23S	2944	11691	14634	0.56
rrnO-23S	2944	11691	14634	0.56
rrnD-23S	2923	948418	951340	0.56
rrnD-23S	2923	948418	951340	0.56
rrnG-23S	2916	173225	176140	0.55
rrnG-23S	2916	173225	176140	0.55
rrnA-23S	2921	32190	35110	0.55
rrnH-23S	2913	168227	171139	0.52
rrnH-23S	2913	168227	171139	0.52
RNase P RNA	382	2331325	2331706	0.12
tRNA peaks with an RPKM ratio of >2				
**Gene**	**Peak Length**	**Coordinates**	**Coordinates**	**RPKM Ratio[a]**
trnY-Glu	230	4155048	4155277	2.76
trnY-Lys	230	4155048	4155277	2.76
trnSL-Gln1	74	2563888	2563961	2.61
trnE-Asp	131	640376	640506	2.35
trnY-Phe	217	4154761	4154977	2.25
trnI-Gly	90	165953	166042	2.12

[a] The expression of each peak (described in the main text) was quantified in reads per kilobase per million mapped reads, or 'RPKM' (Mortazavi *et al*., 2008). The ratio of these values for the Hfq^{FLAG} and mock control samples was taken as an indicator of Hfq-mediated enrichment.
