# Supplementary material for: Association of RNAs with Bacillus subtilis Hfq
Source: PLoS One. 2013 Feb 15;8(2):e55156. doi: 10.1371/journal.pone.0055156 (PMC3574147; doi:10.1371/journal.pone.0055156)
Supplement: Table S2 — Hfq coIP peaks associated with mRNA coding regions. The expression of each peak (described in the main text) was quantified in reads per kilobase per million mapped reads, or ‘RPKM’ [47]. The ratio of these values for the HfqFLAG and mock control samples was taken as an indicator of Hfq-mediated enrichment. Included in this table are data corresponding to peaks that are associated with different regions of mRNA coding regions. (PDF) [file pone.0055156.s006.pdf]

Table S2 Hfq coIP peaks associated with mRNA coding regions.

Peaks That Fully Encompass mRNA Coding Regions			
**Gene**	**Peak Length**	**Peak Coord**	**RPKM Ratio[a]**
yqzF	256	2507015-2507270	36.5
yqgW	214	2565687-2565900	31.4
yhdX	176	1038635-1038810	29.0
yxzF	113	3964110-3964222	24.3
ydzK	191	490817-491007	20.7
yuzK	183	3360983-3361165	18.3
ypmP	226	2292419-2292644	17.1
yxiE	460	4031798-4032257	16.3
yhzC	244	1116582-1116825	11.8
veg	276	52761-53036	10.1
rpsU	158	2620383-2620540	9.00
ywzA	229	3918808-3919036	7.50
sunA	210	2269505-2269714	5.64
ykzF	265	1485069-1485333	4.70
yqzM	160	2637373-2637532	4.70
rsiW	675	195360-196034	3.85
ykzW	110	1534108-1534217	2.94
Peaks Associated With Start Codons			
**Gene**	**Peak Length**	**Peak Coord**	**RPKM Ratio**
yitY	88	1192831-1192918	1200
yvpB	64	3589594-3589657	160
addB	146	1136313-1136458	120
tcyL	152	3006440-3006591	51.1
yheJ	93	1045004-1045096	45.9
yjiA	66	1290892-1290957	26.2
yqgY	82	2564619-2564700	26.0
ypjP	83	2299076-2299158	25.7
glnJ	56	265494-265549	25.4
ctaD	121	1561556-1561676	23.5
yopK	102	2210061-2210162	21.9
yneF	66	1922546-1922611	16.9
minJ	72	3621478-3621549	16.9
nagP	178	840647-840824	13.7
csbA	133	3615078-3615210	11.5
sboA	166	3836036-3836201	3.67
hpr	102	1073612-1073713	2.64
Peaks Associated With Stop Codons			
**Gene**	**Peak length**	**Peak Coord**	**RPKM Ratio**
mecA	106	1229626-1229731	102
fadE	271	3367001-3367271	76.9
yopS	192	2203847-2204038	72.5
yhdT	70	1036862-1036931	71.1
rapB	117	3770985-3771101	32.1
spoIVA	63	2386180-2386242	27.4
hfq	76	1867638-1867713	24.1

tlp	136	1930562-1930697	23.9
sacT	72	3906125-3906196	21.2
ywmB	91	3779267-3779357	20.1
yebD	134	697224-697357	18.9
glpD	103	1006530-1006632	14.8
cccA	136	2599474-2599609	14.2
fbp	79	4130048-4130126	14.1
rnr	109	3451848-3451956	13.9
degU	73	3644596-3644668	13.9
yjbJ	95	1235156-1235250	13.9
ytzI	102	3136927-3137028	11.9
csbA	133	3615078-3615210	11.5
ydbL	221	504809-505029	11.0
ohrB	189	1382249-1382437	10.7
degA	154	1164013-1164166	9.90
hutP	235	4041723-4041957	9.51
clpP	133	3546708-3546840	5.58
yuiB	126	3299316-3299441	5.00
adcA	155	309152-309306	3.67
yqhP	152	2542248-2542475	2.34
citZ	77	2981133-2981209	1.91
Peaks Within mRNA Coding Regions			
**Gene**	**Peak Length**	**Peak Coord**	**RPKM Ratio[a]**
srfAA	162	377729-377890	270
bioD	137	3091835-3091971	217
yitQ	98	1185464-1185561	142
miaA	64	1866987-1867050	108
yvcD	77	3576599-3576675	104
ykuC	228	1476174-1476401	103
truB	97	1737649-1737745	94.3
yqhL	61	2545230-1737745	66.6
yqzK	108	2449501-2449608	63.0
yukB	109	3273707-3273815	55.0
opuBB	89	3461854-3461942	49.1
secDF	60	2828778-2828837	42.3
yerI	54	725248-725301	41.4
gutP	52	668773-668824	41.0
ycbU	80	288459-288538	40.7
srfAB	258	389722-389979	31.3
besA	107	3292960-3293066	31.3
alaT	134	3225984-3226117	30.3
pnpA	102	1739618-1739719	29.9
wapA	54	4025841-4025894	28.2
radC	54	2862144-2862197	26.3
dnaK	89	2627350-2627438	25.1
ydiG	126	646894-647019	25.0
fadE	65	3367929-3367993	23.4
glcD	97	2934073-2934169	23.0
copA	64	3443019-3443082	20.6

ykrW	125	1427086-1427210	18.7
acdA	96	3814111-3814206	18.0
treP	114	851440-851553	17.8
holB	97	40752-40848	17.8
ytrP	71	3034960-3035030	17.2
srfAA	425	386241-386665	17.0
comC	60	2864721-2864780	16.9
yflT	164	827484-827647	16.1
ydaS	94	492709-492802	15.1
sigW	379	194889-195267	14.2
ydaG	225	473804-474028	13.5
ctrA	160	1023440-1023599	13.3
ypjP	159	2298907-2299065	12.1
malP	101	892290-892390	12.1
besA	138	3292745-3292882	11.8
thiF	188	1246288-1246475	11.3
yusK	126	3369240-3369365	10.5
ygzB	155	945000-945154	8.52
dhbF	61	3287221-3287281	7.79
yfmE	66	823759-823824	6.47
gapB	121	2967347-2967467	2.79
yheE	103	1050457-1050559	2.50
citZ	71	2981810-2981880	2.06
ybfO	56	251096-251151	2.06

[a] The expression of each peak was quantified in reads per kilobase per million mapped reads, or 'RPKM' (Mortazavi *et al*., 2008). The ratio of these values for the Hfq^{FLAG} and mock control samples was taken as an indicator of Hfq-mediated enrichment.
