# Supplementary material for: Association of RNAs with Bacillus subtilis Hfq
Source: PLoS One. 2013 Feb 15;8(2):e55156. doi: 10.1371/journal.pone.0055156 (PMC3574147; doi:10.1371/journal.pone.0055156)
Supplement: Table S3 — Hfq coIP peaks associated with mRNA leader regions. The expression of each peak (described in the main text) was quantified in reads per kilobase per million mapped reads, or ‘RPKM’ [47]. The ratio of these values for the HfqFLAG and mock control samples was taken as an indicator of Hfq-mediated enrichment. Included in this table are data corresponding to peaks that are associated with 5' leader regions. (PDF) [file pone.0055156.s007.pdf]

Table S3 mRNA leader regions that coIP with Hfq

Name	Ligand	Peak Length	Peak Coordinates	RPKM Ratio[a]
ilvB	tRNA	76	2897048-2897123	317
yybP	Unknown	101	4169826-4169926	211
mgtE	Magnesium	203	1395622-1395824	130
rplT	Protein	177	2953417-2953593	115
tyrS	tRNA	219	3037940-3038158	88.6
xpt	Purine	135	2320065-2320199	68.3
glpF	Protein	96	1002367-1002462	56.1
ydaO	Unknown	195	486090-486284	41.5
thiU	TPP	172	1391680-1391851	38.7
rplJ	Protein	100	119850-119949	37.6
ykoY	Unknown	130	1410625-1410754	36.9
ptsG	Protein	96	1456978-1457073	31.8
bglP	Protein	121	4035698-4035818	31.6
trpE	Protein	108	2377504-2377611	30.3
rpsD	Protein	118	3035565-3035682	30.1
thrS	tRNA	233	2961244-2961476	28.3
thiT	TPP	123	3179111-3179233	28.1
yitJ	SAM	203	1180645-1180847	27.2
pyrG[b]	Protein	76	3812400-3812475	26.7
trpS	tRNA	223	1219144-1219366	24.1
queC	Purine	82	1439273-1439354	18.6
lysP	Lysine	229	3421133-3421361	18.4
ktrA	Unknown	172	3188194-3188365	18.3
fmnP	FMN	199	2410680-2410878	12.3
yxjG	SAM	123	3999167-3999289	12.2
yxjA	Purine	149	4005557-4005705	11.6
tenA	TPP	140	1242260-1242399	11.2
metQ	SAM	166	3364355-3364520	11.1
thiC	TPP	165	955648-955812	9.92
lysC	Lysine	171	2910880-2911050	5.44
pbuG	Purine	131	694450-694580	4.56
ileS	tRNA	197	1613087-1613283	4.14
gcvT	Glycine	172	2549415-2549586	3.82
ribD	FMN	132	2431468-2431599	3.08
purE	Purine	140	698396-698535	2.93
yoaD	SAM	125	2025141-2025265	2.79
cspC[b]	Protein	45	559567-559611	1.91

[a] The expression of each peak was quantified in reads per kilobase per million mapped reads, or 'RPKM' (Mortazavi et al., 2008). The ratio of these values for the Hfq^{FLAG} and mock control samples was taken as an indicator of Hfq-mediated enrichment.

[b] Almost all of the Hfq CoIP peaks shown in this table directly correspond to the aptamer portions of the cis-acting regulatory RNAs. There are two exceptions – *pyrG* and *cspC*. *pyrG* does not have a discrete aptamer domain according to current literature. Instead, it regulates downstream gene expression through reiterative incorporation of nontemplated G residues under conditions of pyrimidine

limitation, which in turn affects formation of a downstream intrinsic terminator. The Hfq CoIP peak for *pyrG* exactly corresponds to the entire regulatory RNA, from the start of transcription to the transcription terminator element. Therefore, despite the absence of a recognizable aptamer domain, the ligand-sensing portion of the *pyrG* regulatory RNA still corresponds to the Hfq CoIP peak. Expression of *cspB* and *cspC* genes is auto-regulated through a transcription attenuation mechanism (Graumann and Marahiel, 1999). The aptamer portion of these leader regions has not been fully identified; however, the *cspC* Hfq CoIP peak covers the region between the transcription start site and the downstream transcription terminator, and is therefore likely to encompass the putative CspC binding site.
