# Supplementary material for: Association of RNAs with Bacillus subtilis Hfq
Source: PLoS One. 2013 Feb 15;8(2):e55156. doi: 10.1371/journal.pone.0055156 (PMC3574147; doi:10.1371/journal.pone.0055156)
Supplement: Table S4 — Oligonucleotides used in this study. Included in this table are the sequences and brief descriptions of the DNA oligonucleotides used in the experiments described by this manuscript. (PDF) [file pone.0055156.s008.pdf]

Table S4. Oligonucleotides used in this study.

Name	Description	Sequence (written 5’ to 3’)
MD324	Forward oligonucleotide for amplification of *hfq* upstream region	ATCATCGGATCCCAAGAAGAGTCATTCGCGCACTGG
MD325	Reverse oligonucleotide for amplification of *hfq* upstream region. Adds a stop codon and *Not I* site to *hfq* coding sequence.	CGGGCGGCCGCCTACATGTTTCGTCCTCCTTGATTCTC
MD326	Forward oligonucleotide for amplification of *hfq* and its downstream region. Adds stop codon and *Not I* site to *hfq* coding sequence.	ATGTAGGCGGCCGCCCGATTAATATTCAGGATCAGTTT
MD336	Reverse oligonucleotide for amplification of *hfq* and its downstream region. Adds an *EcoRI* site.	AAAAAAGAATTCGAGTTCAAGCTGGACGTTTTTTTG
MD381	Forward oligonucleotide for amplification of *hfq*. Adds the *rpsD* ribosome binding site and a *Hind III* site.	AAAAAAAAGCTTCCAAAGGAGGAGTCACATTATGAAACCGATTAA TATTC
MD382	Reverse oligonucleotide for amplification of *hfq*.Adds *Nhe I* site.	TTTTGCTAGCTTCGAGTTCAAGCTGGACGTTTTTTTG
MD383	Forward oligonucleotide for creation of the sequence encoding for the FLAG epitope. Adds *Nhe I* site.	AAAAGCTAGCGACTACAAAGACCATGACGGTGATTATAAAGATCA TGACATCG
MD384	Forward oligonucleotide for creation of the sequence encoding for the FLAG epitope. Adds *Sph I* site.	TTTTGCATGCTTGTCATCGTCATCCTTGTAATCGATGTCATGATC TTTATAATCACCG
MD395	Forward oligonucleotide for fusion of the FLAG-encoding sequence (3x) to *hfq*. Adds *BamHI* site.	AAAAAAGGATCCATGAAACCGATTAATATTCAG
MD396	Forward oligonucleotide for fusion of the FLAG-encoding sequence (3x) to *hfq*. Adds *EcoRI* site.	TTTTTTGAATTCGCGGCCGCTTACTTGTCATCGTCATCC
MD397	Forward oligonucleotide for amplification of *hfq* downstream region. Adds *EcoR*I site.	AAAAAAGAATTCGCCATGTCAAGACATGAG
MD398	Reverse oligonucleotide for amplification of *hfq* downstream region. Adds *Bgl* II site.	TTTTTTAGATCTGATATAGCCGTCCTCC
MD399	Forward oligonucleotide for amplification of an internal region of *hfq* for the purposes of qPCR quantification.	TCAAATCCGGAAAGAAAATACGTAT
MD400	Reverse oligonucleotide for amplification of an internal region of *hfq* for the purposes of qPCR quantification.	GCCCCGCAACTGAAAGC
MD414	Oligonucleotide used as probe in Northern analysis of the *ykoY* orphan riboswitch region.	GCACCTTGGTGATTTCATTGAA
MD421	Forward oligonucleotide for amplification of an internal region of *sigA* for the purposes of qPCR quantification.	TGCGGCGTGGTCAGAAG
MD422	Reverse oligonucleotide for amplification of an internal region of *sigA* for the purposes of qPCR quantification.	GACGACTTTGTTTCCGGAATACATTCCGGACGCTACTCCCC
MD425	Oligonucleotide used as a probe during Northern blotting analysis of CsfG.	GGAGAAACCGGAGGAAGAACTTATGGGGAAACG
MD426	Oligonucleotide used as a probe for Northern blotting analysis of the orphan riboswitch leader region, *ylbH*.	CGTTTCCCCATAAGTTCTTCCTCCGGTTTCTCC
MD427	Oligonucleotide used as a probe for Northern blotting analysis of *txpA*.	CAAAGCCGATCATGACCATTAGAGATTCATAGGTCG
MD428	Oligonucleotide used as a probe for Northern blotting analysis of *ratA*.	CAAGTGGTAATGTGGTAATGTGGTACCAACTATAAGCTTAC
MD429	Oligonucleotide used as a probe for *ncr1019*.	GCATTAATGCCTGGAATGTTGACATAGCATCACCCC
MD430	Oligonucleotide used as a probe for Northern blotting analysis of *bsrE*.	GTACAGAGCCGGGGTGTTGGTAGCACCTCGGTC
MD433	Oligonucleotide used as a probe for Northern blotting analysis of *bsrG*.	CATTATCATTAATGATTCGTAAACAGTCATTTTCCCACCCC
MD434	Oligonucleotide used as a probe for Northern blotting analysis of *ncr1932*.	GAGCCAGGGTGCTACCAACACCCTGGTCTTTTTATTTTATG
